# Sirtuins in kidney homeostasis and disease: where are we now?

**DOI:** 10.3389/fendo.2024.1524674

**Published:** 2025-01-22

**Authors:** Zhongyu Fan, Xuejiao Wei, Xiaoyu Zhu, Yujun Du

**Affiliations:** Department of Nephrology, The First Hospital of Jilin University, Changchun, China

**Keywords:** sirtuins, chronic kidney disease, acute kidney injury, mitochondria, inflammation, oxidative stress

## Abstract

Sirtuins, identified as (NAD^+^)- dependent class III histone deacetylases, engage in a spectrum of biological functions, encompassing DNA damage repair, oxidative stress, immune modulation, mitochondrial homeostasis, apoptosis and autophagy. Sirtuins play an apoptosis role in regulating cellular operations and overall organism health. Mounting data indicate that dysregulated sirtuin expression is linked to the onset of renal diseases. Effective modulation of sirtuins expression and activity has been shown to improve renal function and attenuate the advancement of kidney diseases. In this review, we present a comprehensive overview of the biological impacts of sirtuins and their molecular targets in regulating renal diseases. Additionally, we detail advancements in elucidating sirtuin roles in the pathophysiology of both chronic and acute renal disorders. We review compounds that modulate sirtuin activity through activation or inhibition, potentially improving outcomes in renal disease. In summary, strategic manipulation of sirtuin activity represents a prospective therapeutic approach for renal diseases.

## 1 Introduction

Sirtuins (SIRTs) constitute a family of evolutionarily conserved (NAD+)- dependent histone deacetylases, pivotal for their enzymatic functions. Sirtuins represent a class III family of (NAD^+^)- dependent histone deacetylases intimately linked to physiological health and disease development across organisms (1). Currently, seven distinct members of the sirtuin family, Sirt1 through Sirt7, have been characterized in mammals. These isoforms share a conserved core domain but exhibit unique active sites that confer specific biological roles (2). Sirtuins are (NAD^+^)- dependent enzymes; their activity converts NAD^+^ into nicotinamide (NAM). Subsequently, NAM is converted to nicotinamide mononucleotide (NMN) by intracellular nicotinamide phosphoribosyltransferase (iNAMPT). NMN is then enzymatically converted to NAD^+^ by nicotinamide mononucleotide adenylyltransferase (NMNAT), the key rate-limiting enzyme in this regeneration cycle, thus perpetuating the sequence (3). NAD^+^ concentrations are intricately linked to disease evolution, with numerous studies demonstrating a decline in NAD^+^ levels concurrent with renal disease advancement. Augmentation of NAD^+^ has been shown to mitigate these effects. The administration of precursors like NAM has been shown to alleviate mild ischemic AKI in both preclinical models and patients (4). Nevertheless, the precise mechanisms underlying the therapeutic benefits of NAD+ supplementation remain incompletely understood.

Over the past two decades, the sirtuin family has garnered significant interest due to their regulatory roles across a spectrum of essential biological mechanisms observed in preclinical and clinical models. These processes include inflammation, oxidative stress, autophagy, mitochondrial homeostasis, deoxyribonucleic acid (DNA) repair, and other crucial functions vital for sustaining cellular and systemic homeostasis (5).

Sirtuin activation has been shown to decelerate the advancement of various renal pathologies, such as acute kidney injury (AKI), diabetic nephropathy (DN), fibrosis and aging. While numerous investigations have established sirtuins as modulators in renal pathology, their precise functions continue to be elucidated. Sirtuins have been identified as potential therapeutic targets across multiple disease states, with small molecules and natural compounds that modulate sirtuin activity emerging as promising candidates for therapy (6). Ongoing investigations into the sirtuin family have facilitated the creation of specific modulators, including curcumin and resveratrol, that enhance kidney health by effecting Sirt1 and Sirt3. Conversely, synthetic inhibitors like AK-1 target sirtuins, specifically inhibiting Sirt2 activity, which has been shown to mitigate the progression of renal diseases (7). This review consolidates research on sirtuin-mediated regulation within renal cells, emphasizing the diverse roles of sirtuin family members and underscoring the therapeutic promise of sirtuin modulators in kidney disease management (6).

## 2 Search strategy

A thorough literature review was conducted across multiple databases, including Embase, PubMed and Web of Science databases, utilizing the following search terms: “SIRT,” “Sirtuins,” “chronic kidney disease,” “acute kidney injury,” and “kidney disease,”. Boolean operators, including OR and AND, were applied in conjunction with the selected search terms to refine the query. Searching for articles from the last ten years. Only English-language articles were included in this review, without restrictions on geographic location.

## 3 The sirtuin family: evolutionary origins and biological functions

**Figure 1:**
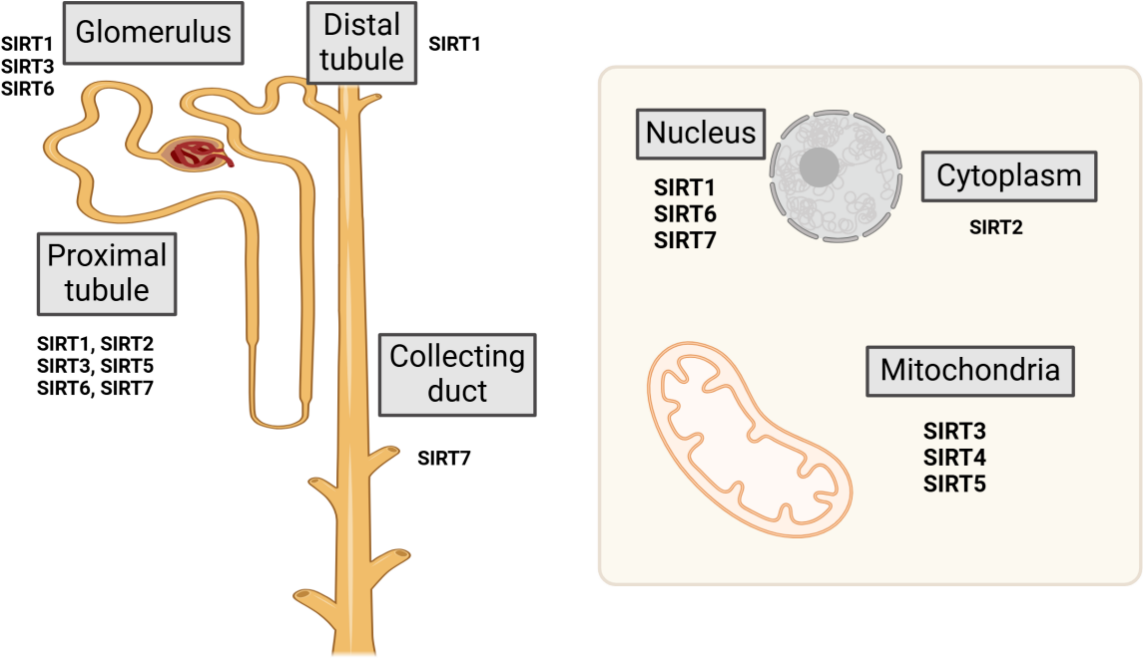
Location and distribution of sirtuins.

Sirtuins represent a highly conserved family of (NAD^+^)-dependent class III histone deacetylases, extensively distributed across both prokaryotic and eukaryotic organisms. The sirtuin family comprises seven homologous proteins, designated as Sirt1 through Sirt7. The members of the sirtuin family are localized to distinct cellular compartments. Sirt1 and Sirt2 are present in both the cytoplasm and nucleus, Sirt6 is confined to the nucleus, Sirt7 is localized to the nucleolus, Sirt3, Sirt4, and Sirt5 are situated within the mitochondria (8) (**Figure 1**). Sirtuins exhibit diverse biological roles that stem from their varied binding affinities and subcellular localizations. Sirtuins such as Sirt1, Sirt2, Sirt3, Sirt5, Sirt6, and Sirt7 demonstrate (NAD^+^)-dependent deacetylase activity, facilitating the deacetylation of both histone and non-histone proteins. In contrast, Sirt4 (9) and Sirt6 possess mono-ADP-ribosyltransferase capabilities (10), whereas Sirt5 functions primarily as a desuccinylase (11). Sirtuins necessitate NAD+ as a catalytic cofactor and can consequently be inhibited by NADH; thus, they exhibit significant sensitivity to the intracellular NAD^+^/NADH balance (12). Sirtuins are fundamental to diverse metabolic and biological processes, including apoptosis, cell survival, proliferation, stress responses, cellular senescence, inflammation.

### 3.1 Nuclear sirtuins

The nuclear sirtuins, specifically SIRT1, SIRT6, and SIRT7, exert a wide range of beneficial effects. In addition to targeting histones, these sirtuins are known to deacetylate a variety of non-histone protein substrates, thereby orchestrating multiple physiological processes.

#### 3.1.1 Sirt1

SIRT1, the most extensively researched member of the sirtuin family, predominantly localizes in the nucleus. It modulates nucleosome histone acetylation and regulates the functionality of various transcription factors (13). Sirt1 is predominantly localized within the nucleus; however, under specific stimuli, it translocates to the cytoplasm. This protein serves a crucial function in regulating diverse biological processes, including mitochondrial metabolic dysfunction (14), inflammation (15), oxidative stress (16), telomere integrity (17), DNA damage response (18), and autophagy (19).

Systemic Sirt1 knockout mice exhibit markedly elevated mitochondrial dysfunction and increased mortality following AKI compared to their wild-type counterparts (20). SIRT1 mediates the deacetylation of both histone and non-histone proteins, performing a key role in preserving cellular homeostasis. Cytoplasmic cortactin plays a crucial role in the stabilization of the actin cytoskeleton (21). SIRT1 safeguards podocytes and facilitates glomerular repair by promoting the deacetylation of cortactin within the nucleus. This deacetylation process is essential for the translocation of acetylated cortactin to the cytoplasm, thereby preserving the integrity of the actin cytoskeleton. SIRT1 enhances high glucose (HG)-induced epithelial-mesenchymal transition (EMT) by deacetylating the transcription factor Yin Yang 1 (YY1) (22). The acetylation of high-mobility group box 1 (HMGB1) is a pivotal step required for its nuclear export, cytoplasmic translocation, and subsequent extracellular secretion in renal cells, a process that exacerbates the progression of renal diseases. SIRT1 deacetylates lysine residues on HMGB1, attenuating subsequent inflammatory signaling pathways (23). In a model of renal fibrosis, both acetylation and p53 expression were elevated; however, SIRT1 mitigated the advancement of ferroptosis by promoting the deacetylation of p53 (24). In aged mice, SIRT1 expression was significantly reduced compared to young mice (5 weeks old), resulting in increased ECM deposition. SIRT1 overexpression, by deacetylating hypoxia-inducible factor-1α (HIF-1α), effectively mitigated hypoxia-induced reactive oxygen species (ROS) production, mitochondrial dysfunction, and ECM protein accumulation, exerting a protective impact on the tubulointerstitial compartment of aged kidneys (25).

SIRT1 alleviates renal inflammation through the deacetylation of nuclear factor-κB (NF-κB) (26), and enhances renal energy metabolism and oxidative stress response by deacetylating forkhead box protein (FOXO) (27) and peroxisome proliferator-activated receptor gamma coactivator-1α (PGC-1α). SIRT1 attenuates renal fibrosis through the deacetylation of Smad3 (28), enhances renal hypoxia tolerance through the deacetylation of HIF-1α (25), and protects against apoptosis in resident kidney cells by deacetylating p53 (29). Moreover, recent studies have demonstrated that SIRT1 enhances renal autophagy through the deacetylation of Beclin1 (19), and cooperates with SIRT3 by activating RelB to enhance mitochondrial biogenesis (30). Consistent with these findings, podocyte-specific Sirt1 knockout mice develop renal fibrosis (31), accompanied by disruption of the actin cytoskeleton (21), activation of the NOD-like Receptor Pyrin Domain Containing 3 (NLRP3) inflammasome (32), and mitochondrial dysfunction in podocytes (33). These pathological changes are notably ameliorated in transgenic mice overexpressing SIRT1 in podocytes (34). Notably, several studies have revealed that claudin-1 expression in podocytes is upregulated and correlates with worsened phenotypes in a mouse model of proteinuric nephropathy with proximal tubule-specific SIRT1 knockout, suggesting that SIRT1 plays a role in the crosstalk between glomerular endothelial cells and tubular epithelial cells. Furthermore, evidence indicates that SIRT1 is intimately connected to endocrine signaling pathways (35). Melatonin, a key hormone produced by the pineal gland, is crucial for regulating redox homeostasis, immune responses, and mitochondrial function. Nevertheless, these effects are partially mediated through the activation of SIRT1 (36). SIRT1 plays a multifaceted role beyond deacetylation, encompassing involvement in ubiquitination, phosphorylation and other critical physiological and pathological mechanisms. Specifically, SIRT1 mediates the dephosphorylation and deacetylation of p65 NF-κB and STAT3, thereby attenuating inflammation, oxidative stress, and EMT in diabetic kidney disease (DKD) (37). In a murine model of unilateral ureteral obstruction (UUO), the activation of SIRT1 signaling was correlated with elevated levels of phosphorylated endothelial nitric oxide synthase (eNOS). Furthermore, SIRT1’s interaction with eNOS contributed to ameliorating renal fibrosis, as assessed by fibrosis scoring in the UUO model (38). In diabetic kidney disease, oxidative stress induces SIRT1 ubiquitination, facilitating its degradation. Conversely, inhibiting SIRT1 ubiquitination enhances FoxO3a nuclear translocation and mitigates oxidative stress-induced renal injury in DKD murine models (39).

#### 3.1.2 Sirt6

SIRT6 is a versatile protein that modulates a diverse range of cellular processes. Early studies of its enzymatic activity identified SIRT6 as a mono-ADP-ribosyltransferase and a deacetylase, capable of removing acetyl groups from histone and non-histone substrates alike (40). SIRT6 is defined by its deacetylase activity that is approximately 1000-fold slower than that of other SIRT family members, distinguishing it from its counterparts (41). Notably, the unique structural configuration of SIRT6 facilitates high-affinity binding to NAD^+^ even without the presence of an acetylated substrate. This unique characteristic arises from its diverged zinc-binding domain and a robust single helix that facilitates NAD^+^ binding (41). Characterized by numerous interaction partners and the capacity to catalyze the removal of diverse post-translational modifications, SIRT6 has a critical impact on essential cellular processes, consisting of DNA repair, gene regulation, telomere maintenance, and cell division (42).

SIRT6 also works in synergy with SIRT1, which deacetylates SIRT6 at lysine 33. This deacetylated form of SIRT6 is then anchored to γH2AX, facilitating its retention within the local chromatin and enabling chromatin remodeling (43). SIRT6 possesses three catalytic activities: deacylation, deacetylation and mono-ADP-ribosylation. Among its histone substrates, SIRT6 commonly deacetylates histone H3 at 56 (H3K56) and lysines 9 (H3K9) (44). Sirt6 is essential for telomere integrity, functioning through the deacetylation of histone H3K9, which mitigates telomeric DNA damage and delays the onset of cellular senescence (45). The deacetylation of histone H3K56 modulates the expression of β-catenin target genes, suppresses transcription of genes associated with fibrosis, and influences renal interstitial fibrosis (46). Additionally, Sirt6 extends its deacetylase activity to non-histone substrates within both the nucleus and cytoplasm, targeting proteins such as members of the p53, FOXO family, NAMPT and Smad (47). SIRT6 regulates renal interstitial fibrosis through the deacetylation of runt-related transcription factor 2 (Runx2), facilitating its nuclear export, and triggering the activation of the ubiquitin-proteasome pathway, which results in Runx2 degradation. This process ultimately suppresses vascular calcification in chronic kidney disease (CKD) (48). Moreover, Sirt6 undergoes acetylation by Sirt1, and the two proteins function in concert to preserve organismal homeostasis (49). In the glomeruli of individuals with hypertensive nephropathy, elevated DNA double-strand breaks (DSBs) are associated with reduced Sirt6 expression. Conversely, Sirt6 overexpression, which upregulates nuclear factor-erythroid 2-related factor 2 (Nrf2) and heme oxygenase-1 (HO-1), suppresses angiotensin II(Ang II)-induced ROS production and DNA DSBs, thereby playing a crucial role in mitigating oxidative DNA damage triggered by Ang II stimulation (50). Renal interstitial fibrosis represents a prevalent pathological feature of CKD. Overexpression of Sirt6 mitigates the progression of this condition in CKD by targeting homeodomain-interacting protein kinase 2, as demonstrated by decreased collagen deposition and downregulation ofα-smooth muscle actin and collagen I (51). Progressive epithelial-mesenchymal transition in the kidneys of db/db mice is linked to the downregulation of Sirt6, with diminished Sirt6 levels contributing to worsening renal pathology, including tubular injury. Further investigations have shown that Sirt6 directly interacts with Smad3, where it deacetylates Smad3, thereby inhibiting its transcriptional activity and nuclear accumulation, offering protection against renal injury in diabetic kidney disease (52). Sirt6 also interacts with saturated fatty acids, particularly palmitic acid, facilitating their export from the nucleus. It induces the deacetylation of acyl-CoA synthetase long-chain 5, thereby enhancing FAO. These findings suggest that Sirt6 extends its metabolic regulatory functions beyond the nucleus, offering new insights into its role in kidney disease (53).

#### 3.1.3 Sirt7

SIRT7 is the sole sirtuin localized within the nucleolus, where its deacetylase activity is essential for the transcription of ribosomal DNA. Sirt7 associates with and deacetylates HMGB1, prompting its translocation to the nucleus and enhancing its role in DNA damage repair. Additionally, Nucleophosmin (NPM), another substrate of Sirt7, undergoes deacetylation by Sirt7, which facilitates its relocation from the nucleolus to the nucleoplasm. In the nucleoplasm, deacetylated NPM associates with ubiquitin ligase, thereby inhibiting p53 ubiquitination and degradation, leading to cell cycle arrest and the preservation of DNA damage repair mechanisms (47). Following DNA damage, ataxia-telangiectasia mutated (ATM) is activated by autophosphorylation; evidence suggests that ATM deacetylation is necessary for its subsequent dephosphorylation. Sirt7 has been shown to deacetylate ATM, thereby limiting its persistent phosphorylation and activation, and consequently facilitating DNA damage repair (54). Conversely, the absence of Sirt7 suppresses NF-κB phosphorylation, diminishes p53 nuclear translocation, and mitigates renal inflammation and tubular damage (55). Sirt7 directly downregulates NF-κB expression, reducing cisplatin-induced acute kidney injury and attenuating apoptosis in renal tubular epithelial cells (56). Overexpression of Sirt7, which counters the observed reduction in Sirt7 levels during hypertensive kidney injury, enhances Krüppel-like factor 15/Nrf2 signaling and significantly mitigates Ang II-induced ferroptosis, renal dysfunction, interstitial fibrosis, and epithelial-mesenchymal transition in mice with hypertensive. These findings suggest that Sirt7 represents a potential therapeutic target for treating hypertensive kidney injury (57). Furthermore, Sirt7-deficient mice exhibit protection against AKI, characterized by phosphorylation of p65 and decreased nuclear translocation, along with diminished inflammatory infiltration of renal cells. This protective impact is further substantiated by diminished proteinuria and decreased markers of renal tubular damage (58).

### 3.2 Cytoplasmic sirtuins

#### 3.2.1 Sirt2

SIRT2 is primarily localized in the cytoplasm, exhibiting deacetylase and demyristoylase activities. It holds significant importance in regulating NF-κB signaling, microtubule dynamics, and adipocyte differentiation through the deacetylation of specific substrates, including p65, alpha-tubulin and the transcription factor FOXO1, respectively (59). Moreover, various proteins, including homeobox transcription factor 10 and 14-3-3 β/γ, are recognized as binding partners of SIRT2, though they are not substrates for its deacetylase activity (60). Additionally, SIRT2 has been implicated in the suppression of basal autophagy through its interaction with autophagy-related gene 7 (61). The diversity of SIRT2 substrates and conjugates highlights its multifaceted role in cellular function. SIRT2 influences metabolic processes within the cytoplasm by responding to NAD levels and regulating proteins integral to metabolic homeostasis, including phosphoglycerate kinase and aldolase. Initially identified as a tubulin deacetylase, SIRT2 has since been shown to interact with and regulate a wide array of both non-histone and histone protein substrates (62). Sirt2 modulates the acetylation of p53 at lysine 382, stabilizing p53 within the nucleus, promoting its transcriptional activity, and exerting a crucial function in the DNA damage response (63). Sirt2 is suggested to associate with the BRCA1-BARD1 complex, deacetylating a conserved lysine residue within the complex to facilitate BRCA1-BARD1 heterodimerization. This modification enhances the complex’s localization to regions of DNA damage, thereby promoting efficient homologous recombination (64). Sirt2 plays a critical role in modulating proinflammatory responses. Its overexpression intensifies cisplatin-induced cellular apoptosis, renal injury, and inflammation, while also enhancing the activation of c-Jun N-terminal kinase (JNK) phosphorylation and p38 (65). Conversely, Sirt2 deficiency mitigates lipopolysaccharide-induced neutrophil and macrophage infiltration, leading to improved renal function (66). During renal ischemia/reperfusion, Sirt2 activation leads to the binding and deacetylation of FOXO3a, facilitating its translocation to the nucleus, which in turn activates caspase-3 and caspase-8, thereby initiating apoptosis. Conversely, inhibition of Sirt2 effectively reverses these processes (67). Sirt2 activity is implicated in the induction and expansion of renal fibroblast activity, in contrast, suppression of Sirt2 attenuates renal fibrosis progression and presents a promising therapeutic strategy for managing CKD (68).

### 3.3 Mitochondrial sirtuins

#### 3.3.1 Sirt3

SIRT3 is predominantly localized within the mitochondrial matrix and serves as the principal regulator of the mitochondrial acetylome, distinguishing it from other mitochondrial sirtuins including SIRT4 and SIRT5 (69). As a representative member of the SIRT family, SIRT3 possesses a conserved enzymatic core (amino acids 126-399) that mediates deacetylation in a (NAD^+^)- dependent manner (70). During the initial stages of renal fibrosis, diminished Sirt3 expression is associated with increased mitochondrial acetylation, and Sirt3-knockout mice display a susceptibility to mitochondrial protein hyperacetylation, leading to exacerbated renal fibrosis (71). Impairment of fatty acid oxidation (FAO) is a key contributor to the progression of renal fibrosis. AKI mice display marked FAO disruption and lipid accumulation, accompanied by elevated ROS production. Moreover, Sirt3 deletion exacerbates FAO dysfunction and renal injury in AKI mice. Further mechanistic investigations suggest that Sirt3 may regulate FAO, facilitate repair, and attenuate renal damage through Adenosine 5’-monophosphate (AMPK) activation (72). Evidence shows that even under normal conditions, SIRT3 knockout mice display pronounced hyperacetylation of various mitochondrial proteins (47). In the context of kidney disease, SIRT3 serves a pivotal function in regulating fatty acid oxidation, deacetylating p53 and superoxide dismutase 2 (SOD2), and mitigating renal damage caused by oxidative stress (73). SIRT3 also deacetylates key proteins such as liver kinase B1(LKB1), SOD2, Cyclophilin D(CypD), Mitochondrial Fusion Protein 2(Mfn2), and PGC-1α to inhibit the opening of the mitochondrial permeability transition pore (mPTP), thereby enhancing mitochondrial function and dynamics (74). Notably, SIRT3 has been implicated in the regulation of early renal development. Studies have shown, in AKI mouse models, SIRT3 knockout exacerbates mitochondrial dysfunction (75), oxidative stress (74), renal impairment, apoptosis, and fibrosis (76). SIRT3 deficiency has been reported to facilitate the mesenchymal transition of tubular epithelial cells, contributing to fibrosis in diabetic kidney disease (77). Additionally, like SIRT1, melatonin has been shown in some studies to activate SIRT3 and ameliorate AKI (78). Recent research has also identified sex-related differences in SIRT3’s susceptibility to ischemia-reperfusion injury (79). Moreover, SIRT3 deficiency can enhance abnormal glycolysis, with glycolytic metabolites downregulating SIRT3 and triggering EMT, thereby exacerbating kidney fibrosis (80). Notably, SIRT3 has also been found to mitigate fibrosis in diabetic kidney disease through the Fibroblast Growth Factor Receptor 1 pathway (81).

#### 3.3.2 Sirt4

Sirt4 modulates the posttranslational modifications of diverse proteins through aliphatic amidase activity, deacetylation and Adenosine Diphosphate (ADP)-ribosylation/nucleotidyltransferase functions, influencing a wide range of biological processes (82). Sirt4 promotes ADP ribosylation and inhibits glutamate dehydrogenase activity, subsequently blocking the conversion of glutamate to α-ketoglutarate in the tricarboxylic acid cycle (83). Furthermore, Sirt4 deficiency results in reduced expression and functionality of the glutamate transporter (84), a factor that may play a more critical role than Sirt4’s deacetylation activity. Sirt4 plays an essential role in preserving mitochondrial function and has been implicated in the development of metabolic disorders, such as diabetic kidney disease. In DKD, Sirt4 mRNA and protein expression are significantly diminished in podocytes exposed to glucose stimulation, with the reduction occurring in a concentration-dependent fashion. Sirt4 deficiency triggers the NLRP3 inflammasome and activation of NF-κB signaling, thereby worsening renal injury (85). Elevated FOXO1 levels and reduced Sirt4 expression have been observed in db/db mice, with FOXO1 overexpression further suppressing Sirt4 and aggravating mitochondrial damage. In contrast, FOXO1 gene silencing enhanced Sirt4 expression and partially recovered mitochondrial function (82).

#### 3.3.3 Sirt5

Sirt5 shows a high affinity for negatively charged acyl groups, including glutarate, succinate, and malonate. It primarily catalyzes lysine acylation but exhibits desuccinylase and deglutarylase activities, with limited deacetylase activity (86). The regulation of Sirt5 is influenced by two key molecules: PGC-1α overexpression increases Sirt5 levels, while AMPK activation decreases its expression (87). Ribose-5-phosphate is essential for nucleotide biosynthesis, and studies have shown that Sirt5 knockdown impairs ribose-5-phosphate production, resulting in persistent and irreversible DNA damage (88). P53 plays an essential role in maintaining genomic stability. Following DNA damage, Sirt5 facilitates the desuccinylation of p53 at lysine 120, consequently inhibiting its activity (89). Increased Sirt5 expression mitigates mitochondrial dysfunction by promoting AMPK phosphorylation, as demonstrated by the preservation of mitochondrial structure, restoration of ATP levels, and deceleration of AKI progression (90). Moreover, Sirt5 plays a crucial role in preserving FAO homeostasis in mitochondria and peroxisomes of renal tubular epithelial cells (RTECs), thereby conferring protection AKI-induced damage (91).

## 4 Sirtuins in renal physiology

The kidney ranks among the most metabolically active organs in the human body, mainly due to its continuous responsibilities in blood filtration, electrolyte and acid–blood pressure regulation, nutrient reabsorption and base equilibrium maintenance. Due to their function as key regulators of cellular metabolism, renal sirtuins play a crucial role in these physiological processes, ensuring adequate energy production across various tubular and glomerular regions. The proximal tubules, responsible for reabsorbing over 80% of the glomerular filtrate, are heavily reliant on active transport processes, which necessitate a higher mitochondrial density compared to collecting ducts and distal tubules (92). Within the kidney, SIRT1 is extensively expressed in podocytes and tubular cells. Its substantial expression in aquaporin 2-positive cells within the rat distal nephron suggests a potential role regulating of homeostasis water and sodium. SIRT1 reduces epithelial sodium reabsorption by associating with methyltransferase and the disruptor of telomeric silencing-1, leading to the suppression of α-subunit transcription of the epithelial sodium channel (ENaC) in duct cells. Importantly, this repressive effect of SIRT1 on the ENaC promoter is not contingent on its deacetylase activity. The repressive action of SIRT1 on the ENaC promoter is independent of its deacetylase function. SIRT1’s ability to modulate water handling and sodium in the kidney may have downstream implications for blood pressure regulation (43).

SIRT3 is poised to play a pivotal role in renal function. Strong evidence links SIRT3 activity to the preservation of mitochondrial energy balance and the enhancement of antioxidant defense mechanisms within both distal tubule compartments and proximal. Mitochondria can dynamically alter their size, quantity, and distribution in response to the specific energy demands of the kidney (82, 93). Mitochondria are highly motile organelles operating within a dynamic network, where their functionality depends on intricate molecular machinery that precisely balances the fission and fusion processes (94). The role of SIRT6 in maintaining renal homeostasis has been recently highlighted in Sirt6-deficient mice, which exhibit significant glomerular damage, particularly in podocytes. This damage includes reduced slit diaphragm protein expression and the effacement of foot processes (95). Evidence further supports that SIRT6 is crucial for preserving glomerular permselectivity to plasma proteins and maintaining podocyte function, as Sirt6 deletion has been shown to exacerbate renal hypertrophy and accelerate the progression of proteinuria (96). Taken together, these results highlight the pivotal role of SIRTs in regulating kidney homeostasis, with profound implications for the initiation and progression of renal diseases (**Figure 1**).

## 5 Sirtuins in kidney disease

**Figure 2:**
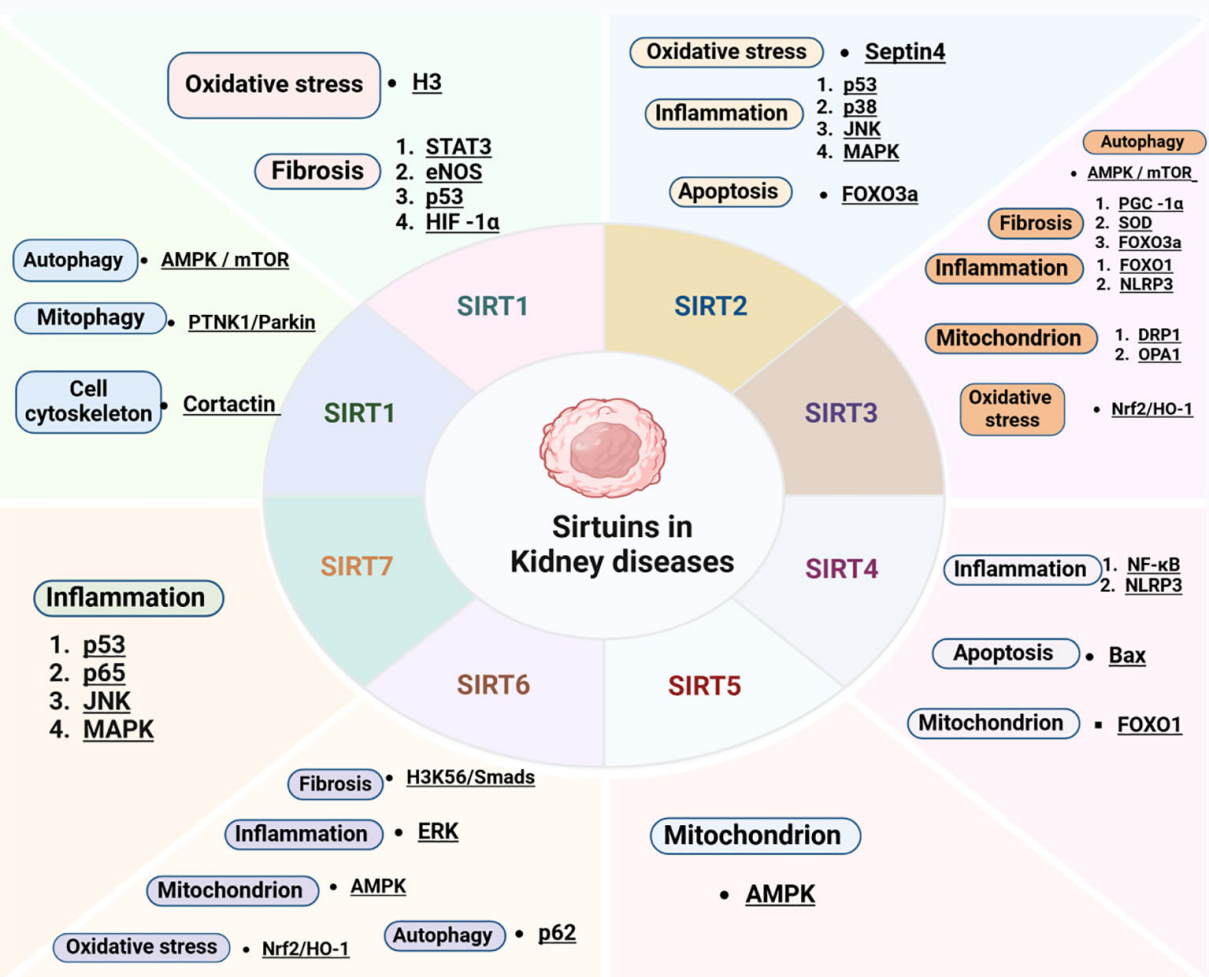
Key molecular targets and cellular mechanisms modulated by sirtuins in renal diseases. HMGB1, high-mobility group box 1; HIF-1α, hypoxia-inducible factor-1; STAT3, signal transducer and activator of transcription 3; YY1, Yin yang 1; eNOS, endothelial nitric oxide synthase; AMPK, AMP-activated protein kinase; mTOR, mammalian target of rapamycin; PINK1, PTEN-induced kinase 1; H3K56, histones3 lysine56; Nrf2, nuclear factor-erythroid 2-related factor 2; HO-1, heme oxygenase-1; ERK, extracellular signal-regulated kinase; NF-κB, nuclear factor kappa B; SOD, superoxide dismutase; PGC-1α, peroxisome proliferator-activated receptor-gamma coactivator 1-alpha; NLRP3, NOD-like Receptor Pyrin Domain Containing 3; DRP1, dynamin-related protein 1; OPA1, optic atrophy 1; JNK, c-Jun N-terminal kinase; MAPK, mitogen-activated protein kinase.

Considering the essential roles of SIRTs in renal diseases, a growing body of research has concentrated on unraveling their influence across a wide range of renal disorders. These discoveries have opened new avenues for identifying innovative therapeutic strategies to slow the progression of renal diseases. Considering the expression of sirtuins in tubular cells and their influence on oxidative stress, inflammation, and mitochondrial dysfunction, they are poised to play a key role in the pathogenesis of AKI (97)(**Figure 2**).

### 5.1 Acute kidney injury

Given their expression in renal tubules and their regulatory influence on oxidative stress, inflammation, and mitochondrial dysfunction, sirtuins are likely fundamental to the development and progression of AKI (97).

#### 5.1.1 Ischemia reperfusion injury

The increased vulnerability of aging kidneys to ischemia/reperfusion (I/R) injury suggests that sirtuins may contribute to the pathogenesis of I/R-induced renal damage. Overexpression of SIRT1 has been linked to increased resistance against kidney injury induced by I/R, whereas the loss of a single SIRT1 allele exacerbated renal damage following I/R (98). SIRT1 mitigated kidney injury caused by I/R by activating antioxidant pathways, including the nuclear factor erythroid Nrf2/HO-1 signaling (99), while also reducing p53 expression and inhibiting apoptosis (98). SIRT1 further mitigated ischemia/reperfusion-induced kidney injury by promoting mitochondrial biogenesis. In the kidney, Ischemia/reperfusion (I/R) injury was observed to upregulate SIRT3 expression. Considering SIRT3’s primary localization within the mitochondrial matrix, it is likely that SIRT3 influences the progression of I/R-induced kidney injury, particularly through its impact on mitochondrial dysfunction. Overexpression of SIRT3 has been demonstrated to offer renal protection by inhibiting superoxide production (100). Diminished SIRT3 expression correlated with heightened severity of I/R-induced renal injury; however, reestablishing SIRT3 levels mitigated this damage by regulating mitochondrial homeostasis via the AMPK/PGC-1α signaling pathway (101). A recent study revealed elevated SIRT5 expression in both peroxisomes and mitochondria of proximal tubular cells. However, unlike other sirtuins, SIRT5 exhibited a divergent role in I/R-induced kidney injury, where its loss conferred renoprotective effects. The proposed mechanism involved the shift of fatty acid oxidation from mitochondria to peroxisomes, regulated by SIRT5. Conversely, SIRT6 expression exhibited an inverse correlation with the severity of hypoxia-induced damage and inflammation in tubular cells (102). The distinct roles of each sirtuin in I/R-induced renal injury warrant further investigation to elucidate their mechanisms and therapeutic potential. In an IRI model of AKI, the deacetylation of PGC1α by SIRT1 was essential for promoting mitochondrial biogenesis and oxidative respiration, thereby sustaining the energy supply necessary for tubular injury after repair (103). Correspondingly, silencing SIRT1 markedly worsened kidney IRI (98).

#### 5.1.2 Cisplatin induced acute kidney injury

Cisplatin-induced renal injury results in decreased mitochondrial quantity and functionality, coupled with increased ROS production. Due to the pivotal role of sirtuins in mitochondrial biogenesis and maintenance, their function has been more thoroughly investigated in the context of cisplatin-induced AKI compared to other etiologies of AKI. Transgenic mice with renal tubule-specific overexpression of SIRT1 exhibited reduced functional and histological indicators of kidney injury following cisplatin administration. This protection was linked to a decrease in cisplatin-induced apoptosis and oxidative stress (104). Recent studies have highlighted the renoprotective function of SIRT3 in cisplatin-induced acute kidney injury through its regulation of mitochondrial dysfunction. In mice models, the absence of SIRT3 function exacerbated renal impairment following cisplatin administration. Conversely, pharmacological activation of SIRT3 alleviated cisplatin-induced renal injury in wild-type mice, but failed to yield the same protective effects in SIRT3-deficient mice. Cisplatin-induced downregulation of SIRT3 led to mitochondrial fragmentation in tubular cells, whereas the restoration of SIRT3 activity counteracted the damage and maintained mitochondrial structural integrity (74). Further studies have reinforced the renoprotective effects of SIRT3 in alleviating cisplatin-induced AKI by modulating mitochondrial dysfunction (105, 106). Among the nuclear sirtuins, SIRT6-deficient mice demonstrated exacerbated cisplatin-induced renal injury. Conversely, SIRT6 mitigates renal apoptosis and inflammation through the deacetylation of H3K9 and the suppression of extracellular signal-regulated kinase (ERK)-1/2 signaling (107).

Contrary to SIRT1, SIRT3, and SIRT6, the deficiency of SIRT2 and SIRT7, rather than their overexpression, notably alleviated cisplatin-induced AKI. This protective effect was achieved by reducing apoptosis and inflammation by modulating JNK and p38 pathways (65, 108). Conflicting evidence exists concerning the function of SIRT5 in cisplatin-induced AKI. One investigation involving renal tubular cells demonstrated that SIRT5 overexpression mitigated cisplatin-induced apoptosis and mitochondrial damage by modulating the B-cell lymphoma 2 (Bcl-2) expression and Nrf2/HO-1 pathway (109). Conversely, another investigation revealed that the absence of SIRT5 function in murine models markedly enhanced renal function and reduced tubular injury in cisplatin-induced AKI via the promotion of peroxisomal fatty acid oxidation in proximal renal tubules (91). The precise function of SIRT5 in cisplatin-induced AKI remains to be fully elucidated.

#### 5.1.3 Other types of acute kidney injury

The protective effects of sirtuins in mitigating sepsis-induced AKI were linked to diminished inflammasome activation and augmented autophagic processes. Nevertheless, in line with observations in cisplatin-induced AKI, the deficiency of SIRT2 in murine models led to enhanced renal function and reduced tubular damage following lipopolysaccharide exposure (65). The role of sirtuins has been further clarified in contrast-induced nephropathy (CIN) (110), which is the third leading cause of hospital-acquired AKI. Reports indicate that oxidative stress, driven by superoxide generation and associated pathways, contributes to the pathogenesis of CIN, with its effects regulated by the expression levels of sirtuins.

##### 5.1.3.1 Hypertensive nephropathy

Growing evidence highlights that SIRTs serve as crucial modulators of renal injury induced by hypertension. Several studies have identified dysregulated expression and activity of SIRTs in models of hypertensive renal damage. In particular, animal models of hypertensive renal injury demonstrated a downregulation of both SIRT1 and SIRT3. An *in vivo* study demonstrated that Ang II administration exacerbates oxidative stress-induced renal damage in aged mice, concomitantly reducing the expression and activity of SIRT1 and SIRT3 (111). Lin et al. additionally observed reduced SIRT3 expression in a murine model of hypertension. Additionally, the single nucleotide polymorphism (SNP) within the regulatory region of the hAT1R gene generates two distinct haplotypes, heteroaryldihydropyrimidine I (Hap-I) and Hap-II (112). Jain et al. demonstrated that transgenic (TG) mice overexpressing the Hap-I variant of the hAT1R gene exhibited enhanced transcriptional activity relative to the Hap-II variant, exacerbating hypertension and chronic renal injury (113). Subsequent investigations revealed that SIRT3 expression was reduced in the renal tissues of aged TG mice expressing the Hap-I variant, implicating SIRT3 in the renal damage associated with hypertension (114).

##### 5.1.3.2 Obesity or hyperlipidemia

Renal lipotoxicity arises from the excessive accumulation of lipids within the kidney (115). Significantly, prior research has indicated that SIRT activation may be crucial in regulating lipid metabolism in AKIs and CKDs. Moreover, restoring lipid metabolism through the modulation of SIRTs could potentially mitigate the progression of these diseases (116). SIRT1 and SIRT3 exert protective functions in preventing disruptions to cellular processes caused by lipotoxicity in renal cells. For example, Wang et al. proposed that SIRT1 restoration can directly counteract apoptosis and mitochondrial dysfunction, as well as lipotoxicity induced by free fatty acids in tubular epithelial cells (TECs) (117). Moreover, overexpression of SIRT3 was shown to mitigate ROS accumulation in the kidney and reduce lipotoxicity-induced inflammation by suppressing the expression of the pro-inflammatory cytokine monocyte chemoattractant protein-1 (MCP-1) (118). Additionally, SIRTs have the potential to regulate fatty acid oxidation in renal cells, thereby protecting against renal damage. For example, rhein has been shown to mitigate renal fibrosis and correct FAO dysfunction by modulating the SIRT1/STAT3/Twist1 axis (119). Activation of SIRT3 has been shown to restore FAO function in tubular epithelial cells and prevent renal fibrosis through the deacetylation of pyruvate dehydrogenase E1α (71). In addition, SIRT5 has been shown to protect against AKI by preserving the equilibrium between mitochondria and FAO in tubular epithelial cells (91). The involvement of SIRTs in lipid metabolism, a contributing factor to kidney diseases, suggests their potential impact on renal injury related to obesity or hyperlipidemia. Moreover, recent research has highlighted the renoprotective role of SIRT3 in mitigating oxidative stress and mitochondrial dysfunction associated with kidney injury induced by a high-fat diet (HFD). Following a high-fat diet (HFD), SIRT3-knockout mice exhibited a greater degree of oxidative stress in the kidneys compared to wild-type mice. This led to significant ultrastructural mitochondrial damage in tubular epithelial cells, decreased energy production and mitochondrial mass, and worsened kidney disease severity (120).

### 5.2 Diabetic nephropathy

Diabetes represents a major public health challenge. It is worth noting that 30–40% of patients with diabetes develop renal complications, such as hyperfiltration, microalbuminuria, diabetic nephropathy, macroalbuminuria, and, eventually, end-stage renal disease (ESRD) (121, 122). However, the pathogenesis of DN is intricate, and the precise molecular mechanisms remain incompletely elucidated. Growing evidence suggests that metabolic oxidative stress, dysregulation, apoptosis, impaired autophagy, and inflammation may contribute to renal pathology and play critical roles in the progression of DN. Recent research has demonstrated that SIRTs are capable of regulating these biological pathways in renal cells, exerting a significant influence on the onset and progression of DN (123, 124). SIRT1 is broadly expressed in human kidneys, and its expression is notably reduced in serum and renal tissues of patients with DN (125). This suppression suggests a strong correlation between SIRT1 levels and renal function. Compelling evidence indicates that SIRT1 plays a renoprotective role in the onset and development of DN (37, 126, 127). *In vivo* studies have also demonstrated that podocyte-specific overexpression of SIRT1 significantly mitigates the progression of DN by reducing diabetes-induced podocyte injury (34). Conversely, conditional knockout of SIRT1 in podocytes led to pronounced proteinuria and severe renal damage in animal models (31). The renoprotective role of SIRT1 has been further validated by *in vitro* studies. For instance, the SIRT1 activator resveratrol has been shown to safeguard podocytes against mitochondrial damage caused by HG concentrations (128). This protective effect is abrogated by the administration of the SIRT1-specific inhibitor EX-527 or by silencing SIRT1 expression (129). Evidence indicates that reduced SIRT1 expression and elevated acetylation of NF-κB p65 may be closely associated with an increased fibrosis index in glomerular mesangial cells (130). In tubular epithelial cells (HK-2) incubated under HG conditions, inhibition of SIRT1 leads to heightened NF-κB activity, resulting in Dynamin-related protein 1(Keap1) overexpression. This process promotes the ubiquitination and subsequent degradation of Nrf2, contributing to renal tubular epithelial injury (131). Upregulation of SIRT1 diminishes HIF-1α expression and activity, leading to the attenuation of renal EMT, fibrosis, and oxidative stress in DN (132, 133). Mounting evidence indicates that the expression of SIRT3, SIRT4, and SIRT7 is reduced in renal cells and tissues affected by DN, implicating their potential renoprotective roles in mammalian kidneys (134).

SIRT3 mitigates HG-induced apoptosis and ROS generation, thereby reducing renal damage in DN by inhibiting Bnip3 (135). Basal autophagy plays a critical role in maintaining homeostasis and biological function in renal cells (136). SIRT3 exerts protective effects against the progression of DN by promoting mitochondrial autophagy (137). As a potential therapeutic approach for DN, SIRT3 was shown to mitigate DN in a diabetic mouse model by facilitating amniotic fluid stem cell-induced protection of mitochondrial homeostasis through the regulation of mitophagy (137). Elevated SIRT3 levels in renal tubular cells have been demonstrated to counteract hyperglycemia-induced apoptosis by reducing ROS accumulation through the activation of the Akt-FoxO signaling pathway (138). Notably, SIRT3 expression is diminished in the kidneys of both mice and patients with DKD (139, 140). Conversely, Sirt3 deficiency has been linked to exacerbated kidney disease in mice subjected to a high-fat diet, a condition that mimics metabolic syndrome in humans (120). Under these conditions, Sirt3 deficiency resulted in lipid accumulation and mitochondrial damage within tubular cells. Ectopic lipid deposition in renal tubules is a prominent pathological hallmark in patients with DKD, arising due to decreased levels of meteorin-like protein (Metrnl), a hormone secreted by skeletal muscle and adipose tissue (141). Under these circumstances, the absence of Sirt3 led to lipid buildup and mitochondrial impairment in tubular cells. This abnormal lipid buildup in renal tubules represents a key pathological characteristic in patients with DKD, arising from reduced levels of meteoric-like protein (Metrnl), a hormone secreted by adipose tissue and skeletal muscle (141).

Previous research demonstrated that SIRT4 mitigates apoptosis via the mitochondrial pathway and suppresses the inflammatory response in DN (142). Overexpression of SIRT4 inhibits podocyte apoptosis by reducing mitochondrial ROS production (142). Additionally, SIRT4 overexpression inhibited the NF-κB signaling pathway by reducing the expression of proinflammatory cytokines and downregulating the NLRP3 inflammasome in podocytes subjected to glucose stimulation (85).

Recent research on the involvement of SIRT2 and SIRT6 in DN has yielded conflicting results. One study reported a reduction in SIRT2 and SIRT6 expression levels under hyperglycemic conditions (125). Conversely, findings from hyperglycemic murine renal podocytes indicate an upregulation of SIRT2 at the mRNA level (143). Nevertheless, SIRT2 mRNA expression declined as the aging process accelerated under hyperglycemic conditions (125). Therefore, the exact biological mechanism of SIRT2 in DN remains to be elucidated.

Some research indicates that SIRT6 may play a pathogenic role in DN. For example, SIRT6 expression has been positively correlated with TNF-α levels, a cytokine linked to systemic inflammation, implying that SIRT6 may exacerbate the inflammatory process in DN (125, 144). Conversely, SIRT6 overexpression may confer protective benefits in DN by modulating mitochondrial dysfunction, glucose metabolism, apoptosis, and the fibrotic phenotype (52, 145). NAD^+^ metabolism is crucial for maintaining kidney function. In mice with proximal renal tubular cell-specific deletion of nicotinamide phosphoribosyltransferase, there is a marked reduction in sirtuin levels, especially Sirt6 (22). Earlier research has demonstrated that FOXO3a safeguards the kidneys against diabetic damage by upregulating Sirt6 expression via the Sirt6/Smad3 signaling pathway (146). The deletion of SIRT6 intensified podocyte damage in diabetic mice, whereas SIRT6 overexpression under high glucose conditions provided protection against podocyte injury by epigenetically regulating the transcription of Notch1 and Notch4 through H3K9 deacetylation (147). SIRT6 has also modulated the immune response by promoting the activation of M2 macrophages, which play a protective role against podocyte damage in STZ-induced diabetic mice (148). A recent investigation revealed that the selective deletion of Nampt in proximal tubular cells of STZ-induced diabetic mice led to decreased SIRT6 expression, which correlated with tubular basement membrane thickening, elevated type IV collagen deposition, aggravated renal fibrosis, and the development of albuminuria (149).

### 5.3 Fibrosis and aging

Renal fibrosis arises from the excessive accumulation of ECM, a progressive condition characterized by tubulointerstitial fibrosis and glomerulosclerosis, which ultimately culminates in end-stage renal disease (150). Renal tubular fibrosis is a key pathological hallmark in CKD, with sirtuins being shown to act as a central factor in its development (151). In a UUO model, SIRT1 knockout mice exhibited marked tubular fibrosis (152). Additionally, the downregulation of SIRT1 in renal medullary interstitial cells significantly diminished their resistance to oxidative stress (153). SIRT1 expression has been identified as a contributing factor in the development of chronic renal allograft dysfunction and chronic cyclosporine A (CsA) nephropathy. In rat kidneys affected by chronic allograft dysfunction, reduced SIRT1 levels are associated with monocyte infiltration and interstitial fibrosis, attributed to the up-regulation of inflammatory cytokines (154). Various mechanisms have been suggested to elucidate the pathogenetic relationship between SIRT1 and the initiation of renal fibrosis. Endothelial SIRT1 expression appears to be critical, as mice with endothelial-specific SIRT1 deletion developed spontaneous interstitial fibrosis, notably in the absence of glomerular involvement, even at an early age. Additionally, after prolonged folic acid treatment, mice with endothelium-specific SIRT1 deletion exhibited exacerbated tubulointerstitial fibrosis (155). Sirtuins have also been implicated in the EMT, a critical mechanism in the advancement of renal fibrosis (156). In alignment with observations from AKI, pharmacological inhibition of SIRT2 led to a reduction in renal interstitial fibrosis in UUO models (68, 157). This effect was accompanied by decreased expression of platelet-derived growth factor receptor-β (PDGFR-β), the E3-ubiquitin ligase murine double-minute 2 (MDM2) (157), epidermal growth factor receptor (EGFR) and signal transducers and activators of transcription 3(STAT3) (68).

Mitochondria are essential in the progression of fibrosis across various organs, including the kidneys. SIRT3 has demonstrated anti-fibrotic properties in cardiac tissue, however, its role in kidney fibrosis remains less well understood (158). Age-related fibrosis was exacerbated in the kidney, likely due to enhanced transforming growth factor-β(TGF-β) signaling and hyperacetylation of glycogen synthase kinase-3β(GSK3β), culminating in the activation of Smad3 in Sirt3-deficient mice (158). Sirt3-deficient mice displayed reduced expression of Opa1 and Mfn1, alongside elevated levels of Drp1, shifting the balance towards mitochondrial fission. This imbalance is linked to renal dysfunction and fibrosis. Additionally, another study revealed that reduced SIRT3 expression is correlated with elevated acetylation in mitochondrial tubular cells during the early stages of renal fibrosis (71).

SIRT3 has been demonstrated to deacetylate kruppel-like factor 15(KLF15), an inhibitor of extracellular matrix protein synthesis, in podocytes. Moreover, in mice with a fibrogenic phenotype, SIRT3 overexpression in endothelial cells provided protection against diabetes-induced renal fibrosis (77). In contrast, Sirt3 deficiency in endothelial cells has been shown to trigger metabolic reprogramming that promotes TGF-β/Smad3-dependent mesenchymal transition in renal tubular epithelial cells (77).

The kidneys are particularly vulnerable to the aging process, which increases their susceptibility to both acute and chronic injuries over time. Preclinical studies have shown that renal SIRT1 activity declines with age, coinciding with a reduction in the intracellular NAD^+^ poo (4), leading to enhanced mitochondrial swelling and disruption of cristae architecture (159). Caloric restriction alleviated mitochondrial abnormalities in aged mice with intact Sirt1, while this benefit was absent in Sirt1-deficient mice (159). Additionally, podocyte-specific deletion of Sirt1 aggravated renal damage and increased cellular senescence in aged mice (27). Similarly, Sirt1 knockout has been linked to the premature onset of endothelial cell senescence (160). Conversely, treatment with a SIRT1 activator or the NAD^+^ precursor nicotinamide mononucleotide has been shown to enhance renal resilience during the aging process in mice (161). Experimental evidence indicates that SIRT1 is pivotal in mediating age-associated pathological alterations, including kidney damage related to aging (162). Prior research has demonstrated a reduction in SIRT1 activity within aging rodent kidneys (163). SIRT1 serves a protective role against apoptosis and senescence triggered by oxidative stress during the aging process (164). It modulates the activity of various FOXO proteins, including FOXO1, FOXO3, and FOXO4, by promoting their deacetylation in response to oxidative stress. Studies have indicated that the PI3K-Akt pathway, which acts as an upstream regulator of FOXO proteins, is downregulated in the aging kidney. The aging process is closely linked to systemic hypoxia, leading to apoptosis, metabolic dysregulation, and cell cycle anomalies. Hypoxia-induced downregulation of SIRT1 expression facilitates FOXO3 acetylation, thereby suppressing the expression of key FOXO3 target genes, including Bnip3 and p27Kip1. Moreover, hypoxia triggers apoptosis and impairs autophagic processes in senescent cells, resulting in the accumulation of these aged cells. In the kidney, SIRT1 safeguards renal cells from apoptosis by preventing Smad7 acetylation through p300 mediation (164). Collectively, these observations underscore the pivotal role of SIRT1 in the progression of age-associated CKD. Calorie restriction has also been demonstrated to upregulate SIRT6 expression, mitigate NF-κB signaling, and enhance renal function in aged mice (114). Research has revealed that the acetylation of nucleophosmin (NPM1) is significantly elevated in senescent cells, accompanied by a pronounced downregulation of SIRT6 and SIRT7, suggesting that these sirtuins may play crucial roles in the aging process via the deacetylation of NPM1. Proteomic analyses of the interaction networks of SIRT6 and SIRT7 have further uncovered potential mechanistic links to aging through their associated protein interaction pathways (165).

### 5.4 Sirtuin regulators

**Figure 3:**
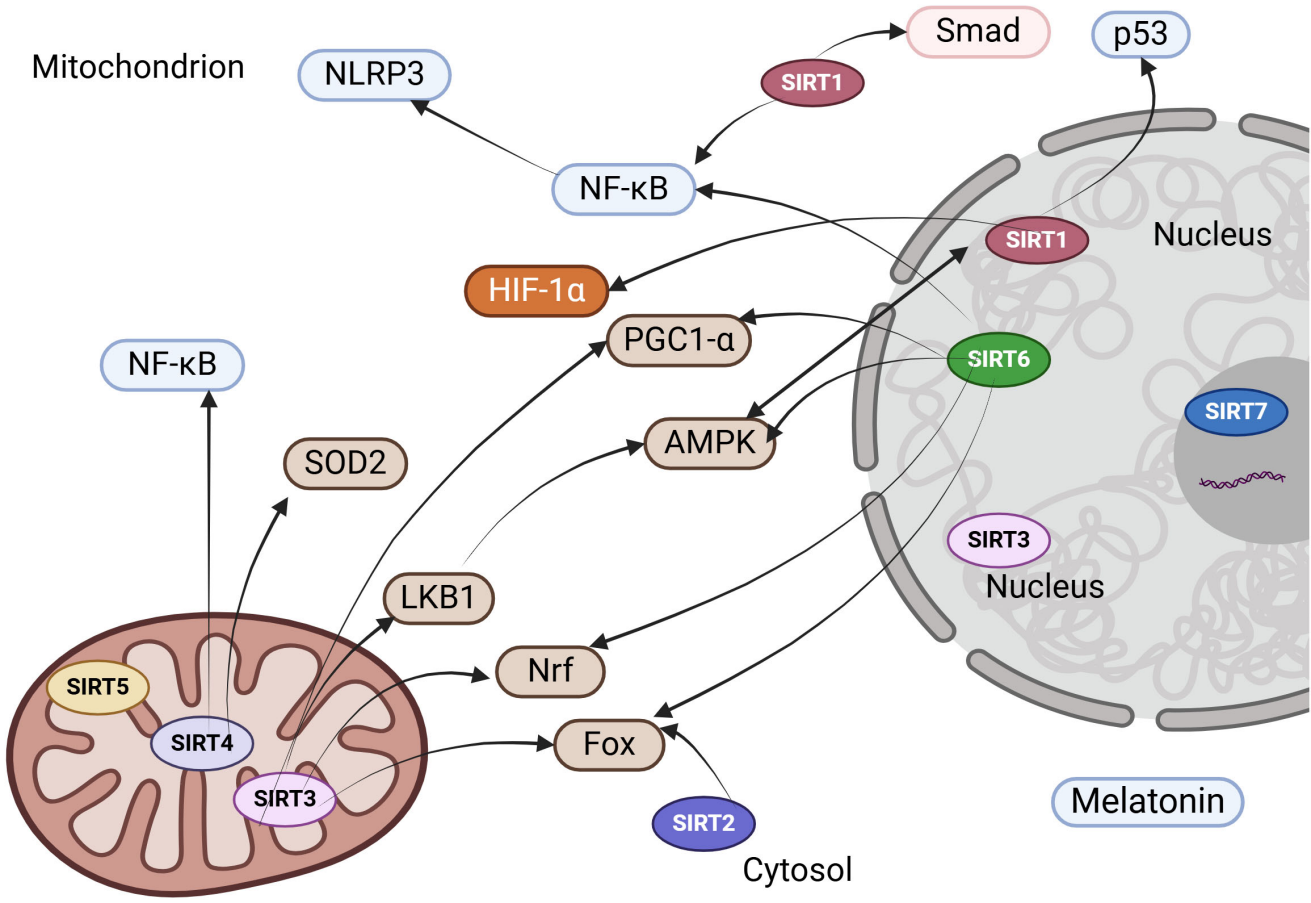
Therapeutic strategies to enhance sirtuin expression and functionality in kidney disease. NLRP3, NOD-like Receptor Pyrin Domain Containing 3; NF-κB, nuclear factor kappa B; HIF-1α, hypoxia-inducible factor-1alpha; AMPK, AMP-activated protein kinase; PGC-1α, peroxisome proliferator-activated receptor-gamma coactivator 1-alpha; SOD, superoxide dismutase; Nrf2, nuclear factor-erythroid 2-related factor 2; LKB1, liver kinase B1; FOXO, forkhead box protein.

Given the involvement of sirtuins in numerous cellular biological processes within the kidney, they represent promising therapeutic targets for the prevention and treatment of age-associated diseases, including renal disorders. Below is a detailed overview of key sirtuin modulators with the most significant implications for kidney disease (**Table 1**, **Figure 3**).

**Table 1:** Treatment of kidney disease related to sirtuins.

Drug/Target	SIRT family	Model (s)	Disease	Genes and pathways	Mechanism of protection	Reference
Resveratrol	Sirt1	DKD/podocyte cells	DKD	Sirt1/PGC-1α	Attenuate mitochondrial oxidative stress	(128)
Curcumin	Sirt1	Aristolochic acid nephropathy/NRK-52E cells	Aristolochic acid nephropathy	Sirt1/Nrf2/HO-1	Activate the body’s antioxidant capacity and reduce tubular epithelial cell apoptosis	(166)
Silymarin	Sirt3	Cisplatin-induced AKI/HK2 cells	Cisplatin-induced AKI	/	Attenuate mitochondrial dysfunction and apoptosis	(106)
Honokiol	Sirt3	Cisplatin-induced/HK2 cells	Cisplatin-induced AKI	Sirt3/AMPK	Remodeled mitochondrial dynamics	(167)
Quercetin	Sirt1	UUO/NRK-52E cells	Senescence and renal fibrosis	Sirt1/PINK1/Parkin	Attenuate mitophagy	(168)
Isoliquiritigenin	Sirt1	STZ-induced DKD/NRK-52E cells	DKD	Sirt1/MAPKs, Sirt1/Nrf2	Alleviate inflammation and oxidative stress	(169)
	Sirt1	STZ-induced DKD	DKD	Sirt1/NF-κB; Sirt1/NLRP3	Reduce inflammation	(170)
Poricoic acid A	Sirt3	UUO/NRK-49F cells	UUO	Sirt3/Wnt/β-catenin	Ameliorate fibrosis	(171)
SRT1720	Sirt1	DKD/podocyte cells	DKD	Sirt1/NF-κB p65	Anti-autophagy response	(172)
SRT3025	Sirt1	Senescence and renal/NRK-49F cells	Senescence and renal fibrosis	Sirt1/TGF-β	Restrain fibrogenesis	(173)
MDL-800	Sirt6	UUO/HK2 cells	UUO	Sirt6/β-Catenin; TGF-β1/Smad	Reduce inflammation and fibrosis	(174)
SRT2183	Sirt1	UUO/RMIC cells	UUO	Sirt1/COX2	Reduce oxidative stress, apoptosis and fibrosis	(153)
AGK2	Sirt2	IRI/RTECs cells	AKI	Sirt2/FOXO3a	Inhibited apoptosis	(67)
	Sirt2	UUO/NRK-49F cells	UUO	Sirt2/EGFR/PDGFRβ	Ameliorate fibrosis	(68)

NMN, nicotinamide mononucleotide; NR, nicotinamide riboside; RTECs, renal tubular epithelial cells; DKD, diabetic kidney disease; AKI acute kidney injury, STZ streptozotocin, UUO unilateral ureteral obstruction, CKD chronic kidney diseases, HK2 Human Kidney-2, PGC-1α peroxisome proliferator-activated receptor-gamma coactivator 1-alpha, PINK1 PTEN-induced kinase 1, Nrf2 nuclear factor-erythroid 2-related factor 2, HO-1 heme oxygenase-1, AMPK AMP-activated protein kinase, MAPK mitogen-activated protein kinase, NLRP3 NOD-like Receptor Pyrin Domain Containing 3, NF-κB nuclear factor kappa B, TGF-β transforming growth factor beta, COX2 cyclooxygenase-2, NRK-49F cultured renal interstitial fibroblasts, EGFR epidermal growth factor receptor, PDGFRβ platelet-derived growth factor receptor-β, RMIC medullary interstitial.

#### 5.4.1 Natural sirtuin agonists

##### 5.4.1.1 Resveratrol

Resveratrol mitigates proteinuria and decreases malondialdehyde concentrations in diabetic mice, while simultaneously enhancing renal cortical Mn-SOD activity, preventing apoptosis in glomerular podocytes and renal tubular epithelial cells, ameliorating histopathological alterations, and restoring the expression of Sirt1 and PGC-1α in renal tissues of DKD models. Moreover, resveratrol directly attenuates mitochondrial reactive oxygen species (ROS) generation, enhances the functionality of respiratory chain complexes I and III, elevates mitochondrial membrane potential, and suppresses the translocation of cytochrome C from mitochondria into the cytoplasm (128).

##### 5.4.1.2 Curcumin

Curcumin, a polyphenolic compound derived from turmeric, modulates oxidative stress and mitigates mitochondrial injury, thereby delaying the initiation and progression of aristolochic acid-induced nephropathy through the activation of the SIRT1/Nrf2/HO-1 signaling cascade (166). Curcumin further mitigates oxidative damage in renal tubular epithelial cells (RTECs) via the Sirt1/FOXO1 pathway. Theaflavin also displays significant renoprotective properties, preventing CaOx-induced renal damage by restoring the antioxidant defense mechanisms mediated through the miR-128–3p/Sirt1 axis (175).

##### 5.4.1.3 Silymarin

Silymarin, a pharmacological Sirt3 activator, confers protection against cisplatin-induced apoptosis in TECs and AKI by enhancing mitochondrial function (106).

##### 5.4.1.4 Honokiol

Honokiol, a small-molecule polyphenol, restores Sirt3 expression and enhances AMPK activity in RTECs exposed to cisplatin. Additionally, it preserves DRP1 phosphorylation at Ser637, preventing its mitochondrial translocation, thereby averting mitochondrial fragmentation and subsequent cellular damage and apoptosis (167).

##### 5.4.1.5 Quercetin

Quercetin, a flavonoid with potent anti-inflammatory and anti-fibrotic effects, has markedly enhanced renal function in murine models and mitigated lipopolysaccharide-induced damage in RTECs. Cellular and murine models demonstrated that quercetin decreased the phosphorylation of IκBα and p65 following lipopolysaccharide exposure. Further investigations revealed that quercetin exerts protective effects against sepsis-induced AKI by upregulating Sirt1 expression and suppressing NF-κB activation (176). Quercetin has been observed to attenuate senescence in RTECs and mitigate renal fibrosis through the activation of Sirt1/PINK1/Parkin-mediated mitophagy (168).

##### 5.4.1.6 Isoliquiritigenin

Isoliquiritigenin, a naturally occurring flavonoid, exerts protective effects against DKD by mitigating inflammation and oxidative stress in a Sirt1-dependent manner. Molecular docking studies have revealed that isoliquiritigenin directly interacts with Sirt1, modulating the MAPK and Nrf-2 signaling pathways to counteract inflammatory responses and oxidative damage, thereby preventing the progression of renal dysfunction and fibrosis (169). Isoliquiritigenin mitigates inflammatory responses by upregulating Sirt-1 activity and modulating the NF-κB and NLRP3 pathways, leading to reduced collagen accumulation in diabetic kidney disease and safeguarding renal architecture and functionality (170).

##### 5.4.1.7 Poricoic acid A

Poricoic acid A, an anti-fibrotic compound derived from Poria cocos, has been demonstrated to suppress renal fibroblast activation and interstitial fibrosis by enhancing SIRT3 expression and promoting β-catenin K49 deacetylation (171). Uncoupling protein 1, a nuclear-encoded protein situated in the inner mitochondrial membrane, has been demonstrated to mitigate oxidative stress by stabilizing SIRT3. This stabilization reduces EMT and ECM deposition, ultimately alleviating renal interstitial fibrosis (177).

#### 5.4.2 Synthetic sirtuin agonists

Considering the pivotal involvement of sirtuin activation in aging-associated pathologies, particularly renal disorders, numerous sirtuin-targeting compounds with high binding affinities have been developed. Notable examples include SRT1720, SRT3025, and MDL-800. SRT1720, a Sirt1 activator, has been shown to decrease p65 acetylation, promote autophagic processes in high glucose-induced podocyte EMT, alleviate renal fibrosis, and restore kidney function (172). SRT3025, another Sirt1 activator, has been documented to counteract the elevation of collagen synthesis induced by TGF-β1, mitigate glomerulosclerosis and tubulointerstitial fibrosis, and ameliorate both the decline in glomerular filtration rate and the severity of proteinuria (173). SRT2183, another Sirt1 activator, has been shown to enhance the resilience of renal medullary interstitial cells to oxidative stress, while reducing apoptosis and fibrosis in a murine model of UUO-induced kidney injury. This effect is mediated by Sirt1-driven upregulation of cyclooxygenase-2 (COX2) expression in renal medullary interstitial cells (153). MDL-800, a Sirt6 activator, has been reported to alleviate tubulointerstitial inflammation and fibrosis in UUO-induced models. *In vitro* studies demonstrated that MDL-800 suppresses TGF-β1-induced myofibroblast activation and ECM production by modulating Sirt6-dependent β-catenin acetylation and regulating the TGF-β1/Smad signaling pathway (174).

#### 5.4.3 Sirtuins inhibitors

Apart from sirtuin activators, a range of sirtuin inhibitors has been developed to treat various renal pathologies. Research has established the role of Sirt2 in driving inflammation and renal fibrosis, leading to the development of Sirt2 inhibitors, including AK-1 and AGK2. Pre-administration of the Sirt2 inhibitor AGK2 before renal ischemia-reperfusion notably diminished the incidence of apoptotic renal tubular cells and alleviated associated ultrastructural damage (67). Sirt2 activity appears to play a role in the activation and proliferation of renal fibroblasts. The Sirt2 inhibitor AGK2 effectively suppressed fibroblast activation and, to a reduced extent, cell proliferation in a dose- and time-dependent manner, as indicated by decreased expression levels of collagen I, α-smooth muscle actin, and fibronectin (68). AK-1, another Sirt2 inhibitor, enhances Nrf2 activity while suppressing JNK signaling, thereby mitigating oxidative stress (178).

## 6 Perspective

The kidney is a complex organ composed of diverse cell types with intricate intercellular signaling pathways that are highly interconnected and mutually regulatory. Investigating and harnessing this cellular crosstalk could provide deeper insights into the underlying mechanisms of renal disease pathogenesis. Since the discovery of sirtuins, our understanding of this protein family has significantly expanded. Initial studies primarily focused on uncovering critical substrates of SIRT enzymatic function. However, subsequent findings indicate that sirtuins modulate functional networks of target proteins, orchestrating a coordinated physiological response across diverse cellular processes such as oxidative stress, metabolism, genomic stability, and cell survival. Emerging evidence suggests that sirtuins are pivotal in alleviating various stressors in metabolically active organs, such as the kidney, thereby influencing physiological and pathological processes. Regrettably, despite extensive efforts in preclinical research, these endeavors have yielded only a limited number of small-molecule candidates progressing to clinical trials. The application of SIRT modulators from laboratory research to clinical application has been impeded by the scarcity of selective compounds targeting specific SIRT isoforms, as well as the moderate efficacy, restricted bioavailability, alongside suboptimal pharmacokinetic and pharmacodynamic properties of current candidates. Considering sirtuins as promising therapeutic targets for the prevention and treatment of age-related disorders, including renal diseases, and potentially extending the human lifespan, validating the clinical benefits of sirtuin activators would have a profound impact on both clinical practice and public health.

Over the past decade, significant advancements have been achieved in the development of effective and safe sirtuin modulators. Several sirtuin agonists have transitioned from preclinical research to clinical trials, opening new avenues for small-molecule therapeutics targeting sirtuins. While sirtuin activators and NAD^+^ enhancers have demonstrated promising outcomes in preclinical studies, including the improvement of pathological markers in podocytes and renal tubular epithelial cells (RTECs), there is currently no robust evidence to support their efficacy in slowing the progression of human kidney disease or in preventing its onset. Crucially, the pharmacokinetic profiles and therapeutic efficacies of sirtuin-targeting agents in renal pathologies are yet to be defined. Additionally, the molecular mechanisms underlying sirtuin-mediated effects require further clarification, and the long-term safety of these therapeutic agents necessitates comprehensive assessment through extended clinical trials. Renal cell communication, mediated through a complex interplay of small molecules, exosomes, and cytokines, is pivotal in the acute biological responses observed during the initiation and progression of renal pathologies. However, the potential of sirtuins as modulators of these signaling interactions merit more comprehensive investigation to enhance therapeutic strategies.

## Glossary

NAM: nicotinamide
NMN: nicotinamide mononucleotide
iNAMPT: intracellular nicotinamide phosphoribosyltransferase
NMNAT: nicotinamide mononucleotide adenylyltransferase
AKI: acute kidney injury
DN: diabetic nephropathy
YY1: Yin Yang 1
HMGB1: high-mobility group box 1
ECM: extracellular matrix
HIF-1α: hypoxia-inducible factor-1α
ROS: reactive oxygen species
NF-κB: nuclear factor-κB
FOXO: forkhead box protein
PGC-1α: peroxisome proliferator-activated receptor gamma coactivator-1α
DKD: diabetic kidney disease
UUO: unilateral ureteral obstruction
eNOS: endothelial nitric oxide synthase
H3K56: histone H3 lysine 56
H3K9: histone H3 lysine 9
Runx2: runt-related transcription factor 2
CKD: chronic kidney disease
DSBs: double-strand breaks
Nrf2: nuclear factor-erythroid 2-related factor 2
HO-1: heme oxygenase-1
FAO: fatty acid oxidation
NPM: nucleophosmin
ATM: ataxia-telangiectasia mutated
ATG7: autophagy-related gene 7
JNK: c-Jun N-terminal kinase
SOD2: superoxide dismutase 2
LKB1: liver kinase B1
CypD: Cyclophilin D
Mfn2: Mitochondrial Fusion Protein 2
mPTP: mitochondrial permeability transition pore
IRI: ischemia-reperfusion injury
RTECs: renal tubular epithelial cells
ENaC: epithelial sodium channel
CIN: contrast-induced nephropathy
Ang II: angiotensin I
Hap-I: heteroaryldihydropyrimidine I
Hap-II: heteroaryldihydropyrimidine II
SNP: single nucleotide polymorphism
TG: transgenic
MCP-1: monocyte chemoattractant protein-1
HFD: high-fat diet
DN: diabetic nephropathy
ESRD: end-stage renal disease
HG: high glucose
Keap1: Kelch-like ECH-associated protein 1
CsA: cyclosporine A
PDGFR-β: platelet-derived growth factor receptor-β
MDM2: murine double-minute 2
EGFR: epidermal growth factor receptor
STAT3: signal transducers and activators of transcription 3
TGFβ: transforming growth factor-β
GSK3β: glycogen synthase kinase-3β
Drp1: Dynamin-related protein 1
KLF15: kruppel-like factor 15
AT1AR: Anti-AT1 receptor antibody
ROS: reactive oxygen species
COX2: cyclooxygenase-2
ECM: extracellular matrix
NPM1: nucleophosmin

## References

[1] Wątroba M Dudek I Skoda M Stangret A Rzodkiewicz P Szukiewicz D. Sirtuins, epigenetics and longevity. Ageing Res Rev. (2017) 40:11–9. doi: 10.1016/j.arr.2017.08.001 28789901

[2] Yuan H Marmorstein R. Structural basis for sirtuin activity and inhibition. J Biol Chem. (2012) 287:42428–35. doi: 10.1074/jbc.R112.372300 PMC3522243 23086949

[3] Ralto KM Rhee EP Parikh SM. NAD(+) homeostasis in renal health and disease. Nat Rev Nephrol. (2020) 16:99–111. doi: 10.1038/s41581-019-0216-6 31673160 PMC7223841

[4] Guan Y Wang SR Huang XZ Xie QH Xu YY Shang D. Nicotinamide mononucleotide, an NAD(+) precursor, rescues age-associated susceptibility to AKI in a sirtuin 1-dependent manner. J Am Soc Nephrol. (2017) 28:2337–52. doi: 10.1681/ASN.2016040385 PMC5533221 28246130

[5] Covarrubias AJ Perrone R Grozio A Verdin E. NAD(+) metabolism and its roles in cellular processes during ageing. Nat Rev Mol Cell Biol. (2021) 22:119–41. doi: 10.1038/s41580-020-00313-x PMC7963035 33353981

[6] Shen P Deng X Chen Z Ba X Qin K Huang Y. SIRT1: A potential therapeutic target in autoimmune diseases. Front Immunol. (2021) 12:779177. doi: 10.3389/fimmu.2021.779177 34887866 PMC8650132

[7] Huang C Jiang S Gao S Wang Y Cai X Fang J. Sirtuins: Research advances on the therapeutic role in acute kidney injury. Phytomedicine. (2022) 101:154122. doi: 10.1016/j.phymed.2022.154122 35490494

[8] Grootaert MOJ Bennett MR. Sirtuins in atherosclerosis: guardians of healthspan and therapeutic targets. Nat Rev Cardiol. (2022) 19:668–83. doi: 10.1038/s41569-022-00685-x 35354967

[9] Min Z Gao J Yu Y. The roles of mitochondrial SIRT4 in cellular metabolism. Front Endocrinol (Lausanne). (2018) 9:783. doi: 10.3389/fendo.2018.00783 30666234 PMC6330279

[10] Chang AR Ferrer CM Mostoslavsky R. SIRT6, a mammalian deacylase with multitasking abilities. Physiol Rev. (2020) 100:145–69. doi: 10.1152/physrev.00030.2018 PMC7002868 31437090

[11] Poniewierska-Baran A Bochniak O Warias P Pawlik A. Role of sirtuins in the pathogenesis of rheumatoid arthritis. Int J Mol Sci. (2023) 24(2):1532. doi: 10.3390/ijms24021532 36675041 PMC9864987

[12] Levine DC Kuo HY Hong HK Cedernaes J Hepler C Wright AG. NADH inhibition of SIRT1 links energy state to transcription during time-restricted feeding. Nat Metab. (2021) 3:1621–32. doi: 10.1038/s42255-021-00498-1 PMC8688143 34903884

[13] Manjula R Anuja K Alcain FJ. SIRT1 and SIRT2 activity control in neurodegenerative diseases. Front Pharmacol. (2020) 11:585821. doi: 10.3389/fphar.2020.585821 33597872 PMC7883599

[14] Oanh NTK Park YY Cho H. Mitochondria elongation is mediated through SIRT1-mediated MFN1 stabilization. Cell Signal. (2017) 38:67–75. doi: 10.1016/j.cellsig.2017.06.019 28669827

[15] Wu J Hao Z Wang Y Yan D Meng J Ma H. Melatonin alleviates BDE-209-induced cognitive impairment and hippocampal neuroinflammation by modulating microglia polarization via SIRT1-mediated HMGB1/TLR4/NF-κB pathway. Food Chem Toxicol. (2023) 172:113561. doi: 10.1016/j.fct.2022.113561 36566971

[16] Lee JJ Ng SC Hsu JY Liu H Chen CJ Huang CY. Galangin reverses H(2)O(2)-induced dermal fibroblast senescence via SIRT1-PGC-1α/nrf2 signaling. Int J Mol Sci. (2022) 23(3):1387. doi: 10.3390/ijms23031387 35163314 PMC8836071

[17] Palacios JA Herranz D De Bonis ML Velasco S Serrano M Blasco MA. SIRT1 contributes to telomere maintenance and augments global homologous recombination. J Cell Biol. (2010) 191:1299–313. doi: 10.1083/jcb.201005160 PMC3010065 21187328

[18] Dong W Zhang K Gong Z Luo T Li J Wang X. N-acetylcysteine delayed cadmium-induced chronic kidney injury by activating the sirtuin 1-P53 signaling pathway. Chem Biol Interact. (2023) 369:110299. doi: 10.1016/j.cbi.2022.110299 36493885

[19] Deng Z Sun M Wu J Fang H Cai S An S. SIRT1 attenuates sepsis-induced acute kidney injury via Beclin1 deacetylation-mediated autophagy activation. Cell Death Dis. (2021) 12:217. doi: 10.1038/s41419-021-03508-y 33637691 PMC7910451

[20] Labiner HE Sas KM Baur JA Sims CA. Sirtuin 1 deletion increases inflammation and mortality in sepsis. J Trauma Acute Care Surg. (2022) 93:672–8. doi: 10.1097/TA.0000000000003751 PMC10673225 35857031

[21] Motonishi S Nangaku M Wada T Ishimoto Y Ohse T Matsusaka T. Sirtuin1 maintains actin cytoskeleton by deacetylation of cortactin in injured podocytes. J Am Soc Nephrol. (2015) 26:1939–59. doi: 10.1681/ASN.2014030289 PMC4520160 25424328

[22] Du L Qian X Li Y Li XZ He LL Xu L. Sirt1 inhibits renal tubular cell epithelial-mesenchymal transition through YY1 deacetylation in diabetic nephropathy. Acta Pharmacol Sin. (2021) 42:242–51. doi: 10.1038/s41401-020-0450-2 PMC8027604 32555442

[23] Wei S Gao Y Dai X Fu W Cai S Fang H. SIRT1-mediated HMGB1 deacetylation suppresses sepsis-associated acute kidney injury. Am J Physiol Renal Physiol. (2019) 316:F20–f31. doi: 10.1152/ajprenal.00119.2018 30379096

[24] Ye Z Xia Y Li L Li B Chen L Yu W. p53 deacetylation alleviates calcium oxalate deposition-induced renal fibrosis by inhibiting ferroptosis. BioMed Pharmacother. (2023) 164:114925. doi: 10.1016/j.biopha.2023.114925 37236026

[25] Ryu DR Yu MR Kong KH Kim H Kwon SH Jeon JS. Sirt1-hypoxia-inducible factor-1α interaction is a key mediator of tubulointerstitial damage in the aged kidney. Aging Cell. (2019) 18:e12904. doi: 10.1111/acel.2019.18.issue-2 30614190 PMC6413666

[26] Gao R Chen J Hu Y Li Z Wang S Shetty S. Sirt1 deletion leads to enhanced inflammation and aggravates endotoxin-induced acute kidney injury. PloS One. (2014) 9:e98909. doi: 10.1371/journal.pone.0098909 24896770 PMC4045768

[27] Chuang PY Cai W Li X Fang L Xu J Yacoub R. Reduction in podocyte SIRT1 accelerates kidney injury in aging mice. Am J Physiol Renal Physiol. (2017) 313:F621–f628. doi: 10.1152/ajprenal.00255.2017 28615249 PMC5625108

[28] Huang XZ Wen D Zhang M Xie Q Ma L Guan Y. Sirt1 activation ameliorates renal fibrosis by inhibiting the TGF-β/Smad3 pathway. J Cell Biochem. (2014) 115:996–1005. doi: 10.1002/jcb.v115.5 24356887

[29] Liang Y Liu H Zhu J Song N Lu Z Fang Y. Inhibition of p53/miR-34a/SIRT1 axis ameliorates podocyte injury in diabetic nephropathy. Biochem Biophys Res Commun. (2021) 559:48–55. doi: 10.1016/j.bbrc.2021.04.025 33932899

[30] Liu TF Vachharajani V Millet P Bharadwaj MS Molina AJ McCall CE. Sequential actions of SIRT1-RELB-SIRT3 coordinate nuclear-mitochondrial communication during immunometabolic adaptation to acute inflammation and sepsis. J Biol Chem. (2015) 290:396–408. doi: 10.1074/jbc.M114.566349 25404738 PMC4281742

[31] Liu R Zhong Y Li X Chen H Jim B Zhou MM. Role of transcription factor acetylation in diabetic kidney disease. Diabetes. (2014) 63:2440–53. doi: 10.2337/db13-1810 PMC4066331 24608443

[32] Jiang M Zhao M Bai M Lei J Yuan Y Huang S. SIRT1 alleviates aldosterone-induced podocyte injury by suppressing mitochondrial dysfunction and NLRP3 inflammasome activation. Kidney Dis (Basel). (2021) 7:293–305. doi: 10.1159/000513884 34395544 PMC8314781

[33] Chuang PY Xu J Dai Y Jia F Mallipattu SK Yacoub R. *In vivo* RNA interference models of inducible and reversible Sirt1 knockdown in kidney cells. Am J Pathol. (2014) 184:1940–56. doi: 10.1016/j.ajpath.2014.03.016 PMC4076473 24952428

[34] Hong Q Zhang L Das B Li Z Liu B Cai G. Increased podocyte Sirtuin-1 function attenuates diabetic kidney injury. Kidney Int. (2018) 93:1330–43. doi: 10.1016/j.kint.2017.12.008 PMC5967974 29477240

[35] Rasha F Mims BM Castro-Piedras I Barnes BJ Grisham MB Rahman RL. The versatility of sirtuin-1 in endocrinology and immunology. Front Cell Dev Biol. (2020) 8:589016. doi: 10.3389/fcell.2020.589016 33330467 PMC7717970

[36] Hardeland R. Aging, melatonin, and the pro- and anti-inflammatory networks. Int J Mol Sci. (2019) 20(5):1223. doi: 10.3390/ijms20051223 30862067 PMC6429360

[37] Sun HJ Xiong SP Cao X Cao L Zhu MY Wu ZY. Polysulfide-mediated sulfhydration of SIRT1 prevents diabetic nephropathy by suppressing phosphorylation and acetylation of p65 NF-κB and STAT3. Redox Biol. (2021) 38:101813. doi: 10.1016/j.redox.2020.101813 33279869 PMC7718489

[38] Chen L Wang Y Li S Zuo B Zhang X Wang F. Exosomes derived from GDNF-modified human adipose mesenchymal stem cells ameliorate peritubular capillary loss in tubulointerstitial fibrosis by activating the SIRT1/eNOS signaling pathway. Theranostics. (2020) 10:9425–42. doi: 10.7150/thno.43315 PMC7415791 32802201

[39] Li S Lin Z Xiao H Xu Z Li C Zeng J. Fyn deficiency inhibits oxidative stress by decreasing c-Cbl-mediated ubiquitination of Sirt1 to attenuate diabetic renal fibrosis. Metabolism. (2023) 139:155378. doi: 10.1016/j.metabol.2022.155378 36538986

[40] Du J Jiang H Lin H. Investigating the ADP-ribosyltransferase activity of sirtuins with NAD analogues and 32P-NAD. Biochemistry. (2009) 48:2878–90. doi: 10.1021/bi802093g 19220062

[41] Pan PW Feldman JL Devries MK Dong A Edwards AM Denu JM. Structure and biochemical functions of SIRT6. J Biol Chem. (2011) 286:14575–87. doi: 10.1074/jbc.M111.218990 PMC3077655 21362626

[42] Gertler AA Cohen HY. SIRT6, a protein with many faces. Biogerontology. (2013) 14:629–39. doi: 10.1007/s10522-013-9478-8 24213807

[43] Meng F Qian M Peng B Peng L Wang X Zheng K. Synergy between SIRT1 and SIRT6 helps recognize DNA breaks and potentiates the DNA damage response and repair in humans and mice. Elife. (2020) 9:e55828. doi: 10.7554/eLife.55828 32538779 PMC7324161

[44] Huang Z Zhao J Deng W Chen Y Shang J Song K. Identification of a cellularly active SIRT6 allosteric activator. Nat Chem Biol. (2018) 14:1118–26. doi: 10.1038/s41589-018-0150-0 30374165

[45] Grootaert MOJ Finigan A Figg NL Uryga AK Bennett MR. SIRT6 protects smooth muscle cells from senescence and reduces atherosclerosis. Circ Res. (2021) 128:474–91. doi: 10.1161/CIRCRESAHA.120.318353 PMC7899748 33353368

[46] Cai J Liu Z Huang X Shu S Hu X Zheng M. The deacetylase sirtuin 6 protects against kidney fibrosis by epigenetically blocking β-catenin target gene expression. Kidney Int. (2020) 97:106–18. doi: 10.1016/j.kint.2019.08.028 31787254

[47] Ren SC Chen X Gong H Wang H Wu C Li PH. SIRT6 in vascular diseases, from bench to bedside. Aging Dis. (2022) 13:1015–29. doi: 10.14336/AD.2021.1204 PMC9286919 35855341

[48] Li W Feng W Su X Luo D Li Z Zhou Y. SIRT6 protects vascular smooth muscle cells from osteogenic transdifferentiation via Runx2 in chronic kidney disease. J Clin Invest. (2022) 132(1):e150051. doi: 10.1172/JCI150051 34793336 PMC8718147

[49] D’Onofrio N Servillo L Balestrieri ML. SIRT1 and SIRT6 signaling pathways in cardiovascular disease protection. Antioxid Redox Signal. (2018) 28:711–32. doi: 10.1089/ars.2017.7178 PMC5824538 28661724

[50] Fan Y Cheng J Yang Q Feng J Hu J Ren Z. Sirt6-mediated Nrf2/HO-1 activation alleviates angiotensin II-induced DNA DSBs and apoptosis in podocytes. Food Funct. (2021) 12:7867–82. doi: 10.1039/D0FO03467C 34240732

[51] Li X Li W Zhang Z Wang W Huang H. SIRT6 overexpression retards renal interstitial fibrosis through targeting HIPK2 in chronic kidney disease. Front Pharmacol. (2022) 13:1007168. doi: 10.3389/fphar.2022.1007168 36172184 PMC9510922

[52] Wang X Ji T Li X Qu X Bai S. FOXO3a protects against kidney injury in type II diabetic nephropathy by promoting sirt6 expression and inhibiting smad3 acetylation. Oxid Med Cell Longev. (2021) 2021:5565761. doi: 10.1155/2021/5565761 34122724 PMC8172321

[53] Hou T Tian Y Cao Z Zhang J Feng T Tao W. Cytoplasmic SIRT6-mediated ACSL5 deacetylation impedes nonalcoholic fatty liver disease by facilitating hepatic fatty acid oxidation. Mol Cell. (2022) 82:4099–4115.e9. doi: 10.1016/j.molcel.2022.09.018 36208627

[54] Tang M Li Z Zhang C Lu X Tu B Cao Z. SIRT7-mediated ATM deacetylation is essential for its deactivation and DNA damage repair. Sci Adv. (2019) 5:eaav1118. doi: 10.1126/sciadv.aav1118 30944854 PMC6436926

[55] Li G Tang X Zhang S Deng Z Wang B Shi W. Aging-conferred SIRT7 decline inhibits rosacea-like skin inflammation by modulating toll-like receptor 2-NF-κB signaling. J Invest Dermatol. (2022) 142:2580–2590.e6. doi: 10.1016/j.jid.2022.03.026 35413292

[56] Chen G Xue H Zhang X Ding D Zhang S. p53 inhibition attenuates cisplatin-induced acute kidney injury through microRNA-142-5p regulating SIRT7/NF-κB. Ren Fail. (2022) 44:368–80. doi: 10.1080/0886022X.2022.2039195 PMC8890533 35220863

[57] Li XT Song JW Zhang ZZ Zhang MW Liang LR Miao R. Sirtuin 7 mitigates renal ferroptosis, fibrosis and injury in hypertensive mice by facilitating the KLF15/Nrf2 signaling. Free Radic Biol Med. (2022) 193:459–73. doi: 10.1016/j.freeradbiomed.2022.10.320 36334846

[58] Sánchez-Navarro A Martínez-Rojas M Albarrán-Godinez A Pérez-Villalva R Auwerx J de la Cruz A. Sirtuin 7 deficiency reduces inflammation and tubular damage induced by an episode of acute kidney injury. Int J Mol Sci. (2022) 23(5):2573. doi: 10.3390/ijms23052573 35269715 PMC8910458

[59] Teodoro JS Duarte FV Gomes AP Varela AT Peixoto FM Rolo AP. Berberine reverts hepatic mitochondrial dysfunction in high-fat fed rats: a possible role for SirT3 activation. Mitochondrion. (2013) 13:637–46. doi: 10.1016/j.mito.2013.09.002 24041461

[60] Bae NS Swanson MJ Vassilev A Howard BH. Human histone deacetylase SIRT2 interacts with the homeobox transcription factor HOXA10. J Biochem. (2004) 135:695–700. doi: 10.1093/jb/mvh084 15213244

[61] Inoue T Nakayama Y Li Y Matsumori H Takahashi H Kojima H. SIRT2 knockdown increases basal autophagy and prevents postslippage death by abnormally prolonging the mitotic arrest that is induced by microtubule inhibitors. FEBS J. (2014) 281:2623–37. doi: 10.1111/febs.2014.281.issue-11 24712640

[62] Gomes P Fleming Outeiro T Cavadas C. Emerging role of sirtuin 2 in the regulation of mammalian metabolism. Trends Pharmacol Sci. (2015) 36:756–68. doi: 10.1016/j.tips.2015.08.001 26538315

[63] Agborbesong E Zhou JX Li LX Harris PC Calvet JP Li X. Prdx5 regulates DNA damage response through autophagy-dependent Sirt2-p53 axis. Hum Mol Genet. (2023) 32:567–79. doi: 10.1093/hmg/ddac218 PMC9896474 36067023

[64] Minten EV Kapoor-Vazirani P Li C Zhang H Balakrishnan K Yu DS. SIRT2 promotes BRCA1-BARD1 heterodimerization through deacetylation. Cell Rep. (2021) 34:108921. doi: 10.1016/j.celrep.2021.108921 33789098 PMC8108010

[65] Jung YJ Park W Kang KP Kim W. SIRT2 is involved in cisplatin-induced acute kidney injury through regulation of mitogen-activated protein kinase phosphatase-1. Nephrol Dial Transplant. (2020) 35:1145–56. doi: 10.1093/ndt/gfaa042 32240312

[66] Jung YJ Lee AS Nguyen-Thanh T Kim D Kang KP Lee S. SIRT2 regulates LPS-induced renal tubular CXCL2 and CCL2 expression. J Am Soc Nephrol. (2015) 26:1549–60. doi: 10.1681/ASN.2014030226 PMC4483578 25349202

[67] Wang Y Mu Y Zhou X Ji H Gao X Cai WW. SIRT2-mediated FOXO3a deacetylation drives its nuclear translocation triggering FasL-induced cell apoptosis during renal ischemia reperfusion. Apoptosis. (2017) 22:519–30. doi: 10.1007/s10495-016-1341-3 28078537

[68] Ponnusamy M Zhou X Yan Y Tang J Tolbert E Zhao TC. Blocking sirtuin 1 and 2 inhibits renal interstitial fibroblast activation and attenuates renal interstitial fibrosis in obstructive nephropathy. J Pharmacol Exp Ther. (2014) 350:243–56. doi: 10.1124/jpet.113.212076 PMC4109489 24833701

[69] Shimazu T Hirschey MD Hua L Dittenhafer-Reed KE Schwer B Lombard DB. SIRT3 deacetylates mitochondrial 3-hydroxy-3-methylglutaryl CoA synthase 2 and regulates ketone body production. Cell Metab. (2010) 12:654–61. doi: 10.1016/j.cmet.2010.11.003 PMC3310379 21109197

[70] Zhang S Ma Y Feng J. Neuroprotective mechanisms of ϵ-viniferin in a rotenone-induced cell model of Parkinson’s disease: significance of SIRT3-mediated FOXO3 deacetylation. Neural Regener Res. (2020) 15:2143–53. doi: 10.4103/1673-5374.282264 PMC7716051 32394973

[71] Zhang Y Wen P Luo J Ding H Cao H He W. Sirtuin 3 regulates mitochondrial protein acetylation and metabolism in tubular epithelial cells during renal fibrosis. Cell Death Dis. (2021) 12:847. doi: 10.1038/s41419-021-04134-4 34518519 PMC8437958

[72] Li M Li CM Ye ZC Huang J Li Y Lai W. Sirt3 modulates fatty acid oxidation and attenuates cisplatin-induced AKI in mice. J Cell Mol Med. (2020) 24:5109–21. doi: 10.1111/jcmm.15148 PMC7205836 32281286

[73] Ouyang J Zeng Z Fang H Li F Zhang X Tan W. SIRT3 inactivation promotes acute kidney injury through elevated acetylation of SOD2 and p53. J Surg Res. (2019) 233:221–30. doi: 10.1016/j.jss.2018.07.019 30502252

[74] Morigi M Perico L Rota C Longaretti L Conti S Rottoli D. Sirtuin 3-dependent mitochondrial dynamic improvements protect against acute kidney injury. J Clin Invest. (2015) 125:715–26. doi: 10.1172/JCI77632 PMC4319434 25607838

[75] Fan H Le JW Sun M Zhu JH. Sirtuin 3 deficiency promotes acute kidney injury induced by sepsis via mitochondrial dysfunction and apoptosis. Iran J Basic Med Sci. (2021) 24:675–81. doi: 10.22038/ijbms.2021.54905.12312 PMC8244614 34249270

[76] Li N Zhang J Yan X Zhang C Liu H Shan X. SIRT3-KLF15 signaling ameliorates kidney injury induced by hypertension. Oncotarget. (2017) 8:39592–604. doi: 10.18632/oncotarget.17165 PMC5503635 28465484

[77] Srivastava SP Li J Takagaki Y Kitada M Goodwin JE Kanasaki K. Endothelial SIRT3 regulates myofibroblast metabolic shifts in diabetic kidneys. iScience. (2021) 24:102390. doi: 10.1016/j.isci.2021.102390 33981977 PMC8086030

[78] Zhang C Suo M Liu L Qi Y Zhang C Xie L. Melatonin alleviates contrast-induced acute kidney injury by activation of sirt3. Oxid Med Cell Longev. (2021) 2021:6668887. doi: 10.1155/2021/6668887 34122726 PMC8169261

[79] Shen H Holliday M Sheikh-Hamad D Li Q Tong Q Hamad CD. Sirtuin-3 mediates sex differences in kidney ischemia-reperfusion injury. Transl Res. (2021) 235:15–31. doi: 10.1016/j.trsl.2021.03.015 33789208

[80] Tan C Gu J Li T Chen H Liu K Liu M. Inhibition of aerobic glycolysis alleviates sepsis−induced acute kidney injury by promoting lactate/Sirtuin 3/AMPK−regulated autophagy. Int J Mol Med. (2021) 47(3):19. doi: 10.3892/ijmm.2021.4852 33448325 PMC7849980

[81] Srivastava SP Goodwin JE Kanasaki K Koya D. Metabolic reprogramming by N-acetyl-seryl-aspartyl-lysyl-proline protects against diabetic kidney disease. Br J Pharmacol. (2020) 177:3691–711. doi: 10.1111/bph.v177.16 PMC7393199 32352559

[82] Pande S Raisuddin S. Molecular and cellular regulatory roles of sirtuin protein. Crit Rev Food Sci Nutr. (2023) 63:9895–913. doi: 10.1080/10408398.2022.2070722 35510883

[83] Yin X Peng J Gu L Liu Y Li X Wu J. Targeting glutamine metabolism in hepatic stellate cells alleviates liver fibrosis. Cell Death Dis. (2022) 13:955. doi: 10.1038/s41419-022-05409-0 36376267 PMC9663710

[84] Shih J Liu L Mason A Higashimori H Donmez G. Loss of SIRT4 decreases GLT-1-dependent glutamate uptake and increases sensitivity to kainic acid. J Neurochem. (2014) 131:573–81. doi: 10.1111/jnc.2014.131.issue-5 25196144

[85] Xu X Zhang L Hua F Zhang C Zhang C Mi X. FOXM1-activated SIRT4 inhibits NF-κB signaling and NLRP3 inflammasome to alleviate kidney injury and podocyte pyroptosis in diabetic nephropathy. Exp Cell Res. (2021) 408:112863. doi: 10.1016/j.yexcr.2021.112863 34626587

[86] Yang L Ma X He Y Yuan C Chen Q Li G. Sirtuin 5: a review of structure, known inhibitors and clues for developing new inhibitors. Sci China Life Sci. (2017) 60:249–56. doi: 10.1007/s11427-016-0060-7 27858336

[87] Buler M Aatsinki SM Izzi V Uusimaa J Hakkola J. SIRT5 is under the control of PGC-1α and AMPK and is involved in regulation of mitochondrial energy metabolism. FASEB J. (2014) 28:3225–37. doi: 10.1096/fj.13-245241 24687991

[88] Wang HL Chen Y Wang YQ Tao EW Tan J Liu QQ. Sirtuin5 protects colorectal cancer from DNA damage by keeping nucleotide availability. Nat Commun. (2022) 13:6121. doi: 10.1038/s41467-022-33903-8 36253417 PMC9576705

[89] Liu X Rong F Tang J Zhu C Chen X Jia S. Repression of p53 function by SIRT5-mediated desuccinylation at Lysine 120 in response to DNA damage. Cell Death Differ. (2022) 29:722–36. doi: 10.1038/s41418-021-00886-w PMC8989948 34642466

[90] Wang T Lin B Qiu W Yu B Li J An S. Adenosine monophosphate-activated protein kinase phosphorylation mediated by sirtuin 5 alleviates septic acute kidney injury. Shock. (2023) 59:477–85. doi: 10.1097/SHK.0000000000002073 36533528

[91] Chiba T Peasley KD Cargill KR Maringer KV Bharathi SS Mukherjee E. Sirtuin 5 regulates proximal tubule fatty acid oxidation to protect against AKI. J Am Soc Nephrol. (2019) 30:2384–98. doi: 10.1681/ASN.2019020163 PMC6900790 31575700

[92] Xu Y Liu L Nakamura A Someya S Miyakawa T Tanokura M. Studies on the regulatory mechanism of isocitrate dehydrogenase 2 using acetylation mimics. Sci Rep. (2017) 7:9785. doi: 10.1038/s41598-017-10337-7 28852116 PMC5575304

[93] Chuang PY Dai Y Liu R He H Kretzler M Jim B. Alteration of forkhead box O (foxo4) acetylation mediates apoptosis of podocytes in diabetes mellitus. PloS One. (2011) 6:e23566. doi: 10.1371/journal.pone.0023566 21858169 PMC3157434

[94] Picard F Kurtev M Chung N Topark-Ngarm A Senawong T MaChado De Oliveira R. Sirt1 promotes fat mobilization in white adipocytes by repressing PPAR-gamma. Nature. (2004) 429:771–6. doi: 10.1038/nature02583 PMC2820247 15175761

[95] Guo H Bechtel-Walz W. The interplay of autophagy and oxidative stress in the kidney: what do we know? Nephron. (2023) 147:627–42. doi: 10.1159/000531290 37442108

[96] Lee IH. Mechanisms and disease implications of sirtuin-mediated autophagic regulation. Exp Mol Med. (2019) 51:1–11. doi: 10.1038/s12276-019-0299-y PMC6802627 31492861

[97] Wakino S Hasegawa K Itoh H. Sirtuin and metabolic kidney disease. Kidney Int. (2015) 88:691–8. doi: 10.1038/ki.2015.157 PMC4593995 26083654

[98] Fan H Yang HC You L Wang YY He WJ Hao CM. The histone deacetylase, SIRT1, contributes to the resistance of young mice to ischemia/reperfusion-induced acute kidney injury. Kidney Int. (2013) 83:404–13. doi: 10.1038/ki.2012.394 23302720

[99] Shi S Lei S Tang C Wang K Xia Z. Melatonin attenuates acute kidney ischemia/reperfusion injury in diabetic rats by activation of the SIRT1/Nrf2/HO-1 signaling pathway. Biosci Rep. (2019) 39(1):BSR20181614. doi: 10.1042/BSR20181614 30578379 PMC6331666

[100] Pan JS Huang L Belousova T Lu L Yang Y Reddel R. Stanniocalcin-1 inhibits renal ischemia/reperfusion injury via an AMP-activated protein kinase-dependent pathway. J Am Soc Nephrol. (2015) 26:364–78. doi: 10.1681/ASN.2013070703 PMC4310644 25012175

[101] Peerapanyasut W Kobroob A Palee S Chattipakorn N Wongmekiat O. Bisphenol A aggravates renal ischemia-reperfusion injury by disrupting mitochondrial homeostasis and N-acetylcysteine mitigates the injurious outcomes. IUBMB Life. (2020) 72:758–70. doi: 10.1002/iub.v72.4 31587481

[102] Gao Z Chen X Fan Y Zhu K Shi M Ding G. Sirt6 attenuates hypoxia-induced tubular epithelial cell injury via targeting G2/M phase arrest. J Cell Physiol. (2020) 235:3463–73. doi: 10.1002/jcp.v235.4 PMC13482674 31603249

[103] Funk JA Schnellmann RG. Accelerated recovery of renal mitochondrial and tubule homeostasis with SIRT1/PGC-1α activation following ischemia-reperfusion injury. Toxicol Appl Pharmacol. (2013) 273:345–54. doi: 10.1016/j.taap.2013.09.026 PMC3987743 24096033

[104] Hasegawa K Wakino S Yoshioka K Tatematsu S Hara Y Minakuchi H. Kidney-specific overexpression of Sirt1 protects against acute kidney injury by retaining peroxisome function. J Biol Chem. (2010) 285:13045–56. doi: 10.1074/jbc.M109.067728 PMC2857112 20139070

[105] Ortega-Domínguez B Aparicio-Trejo OE García-Arroyo FE León-Contreras JC Tapia E Molina-Jijón E. Curcumin prevents cisplatin-induced renal alterations in mitochondrial bioenergetics and dynamic. Food Chem Toxicol. (2017) 107:373–85. doi: 10.1016/j.fct.2017.07.018 28698153

[106] Li Y Ye Z Lai W Rao J Huang W Zhang X. Activation of sirtuin 3 by silybin attenuates mitochondrial dysfunction in cisplatin-induced acute kidney injury. Front Pharmacol. (2017) 8:178. doi: 10.3389/fphar.2017.00178 28424621 PMC5380914

[107] Li Z Xu K Zhang N Amador G Wang Y Zhao S. Overexpressed SIRT6 attenuates cisplatin-induced acute kidney injury by inhibiting ERK1/2 signaling. Kidney Int. (2018) 93:881–92. doi: 10.1016/j.kint.2017.10.021 29373150

[108] Miyasato Y Yoshizawa T Sato Y Nakagawa T Miyasato Y Kakizoe Y. Sirtuin 7 deficiency ameliorates cisplatin-induced acute kidney injury through regulation of the inflammatory response. Sci Rep. (2018) 8:5927. doi: 10.1038/s41598-018-24257-7 29651144 PMC5897539

[109] Li W Yang Y Li Y Zhao Y Jiang H. Sirt5 attenuates cisplatin-induced acute kidney injury through regulation of nrf2/HO-1 and bcl-2. BioMed Res Int. (2019) 2019:4745132. doi: 10.1155/2019/4745132 31815138 PMC6878818

[110] Kim JE Bae SY Ahn SY Kwon YJ Ko GJ. The role of nuclear factor erythroid-2-related factor 2 expression in radiocontrast-induced nephropathy. Sci Rep. (2019) 9:2608. doi: 10.1038/s41598-019-39534-2 30796317 PMC6384919

[111] Pushpakumar S Ren L Juin SK Majumder S Kulkarni R Sen U. Methylation-dependent antioxidant-redox imbalance regulates hypertensive kidney injury in aging. Redox Biol. (2020) 37:101754. doi: 10.1016/j.redox.2020.101754 33080442 PMC7575806

[112] Lin JR Zheng YJ Zhang ZB Shen WL Li XD Wei T. Suppression of endothelial-to-mesenchymal transition by SIRT (Sirtuin) 3 alleviated the development of hypertensive renal injury. Hypertension. (2018) 72:350–60. doi: 10.1161/HYPERTENSIONAHA.118.10482 29915018

[113] Jain S Rana A Jain K Perla SK Puri N Kumar A. Age-related expression of human AT1R variants and associated renal dysfunction in transgenic mice. Am J Hypertens. (2018) 31:1234–42. doi: 10.1093/ajh/hpy121 PMC6454504 30084918

[114] Zhang N Li Z Mu W Li L Liang Y Lu M. Calorie restriction-induced SIRT6 activation delays aging by suppressing NF-κB signaling. Cell Cycle. (2016) 15:1009–18. doi: 10.1080/15384101.2016.1152427 PMC4889297 26940461

[115] Wahl P Ducasa GM Fornoni A. Systemic and renal lipids in kidney disease development and progression. Am J Physiol Renal Physiol. (2016) 310:F433–45. doi: 10.1152/ajprenal.00375.2015 PMC4971889 26697982

[116] Gao Z Chen X. Fatty acid β-oxidation in kidney diseases: perspectives on pathophysiological mechanisms and therapeutic opportunities. Front Pharmacol. (2022) 13:805281. doi: 10.3389/fphar.2022.805281 35517820 PMC9065343

[117] Wang Y He W Wei W Mei X Yang M Wang Y. Exenatide attenuates obesity-induced mitochondrial dysfunction by activating SIRT1 in renal tubular cells. Front Endocrinol (Lausanne). (2021) 12:622737. doi: 10.3389/fendo.2021.622737 34434166 PMC8380782

[118] Koyama T Kume S Koya D Araki S Isshiki K Chin-Kanasaki M. SIRT3 attenuates palmitate-induced ROS production and inflammation in proximal tubular cells. Free Radic Biol Med. (2011) 51:1258–67. doi: 10.1016/j.freeradbiomed.2011.05.028 21664458

[119] Song X Du Z Yao Z Tang X Zhang M. Rhein improves renal fibrosis by restoring cpt1a-mediated fatty acid oxidation through sirT1/STAT3/twist1 pathway. Molecules. (2022) 27(7):2344. doi: 10.3390/molecules27072344 35408745 PMC9000220

[120] Locatelli M Macconi D Corna D Cerullo D Rottoli D Remuzzi G. Sirtuin 3 deficiency aggravates kidney disease in response to high-fat diet through lipotoxicity-induced mitochondrial damage. Int J Mol Sci. (2022) 23(15):8345. doi: 10.3390/ijms23158345 35955472 PMC9368634

[121] Gembillo G Visconti L Giuffrida AE Labbozzetta V Peritore L Lipari A. Role of zinc in diabetic kidney disease. Nutrients. (2022) 14(7):1353. doi: 10.3390/nu14071353 35405968 PMC9003285

[122] Espinel E Agraz I Ibernon M Ramos N Fort J Serón D. Renal biopsy in type 2 diabetic patients. J Clin Med. (2015) 4:998–1009. doi: 10.3390/jcm4050998 26239461 PMC4470212

[123] Tang J Yao D Yan H Chen X Wang L Zhan H. The role of microRNAs in the pathogenesis of diabetic nephropathy. Int J Endocrinol. (2019) 2019:8719060. doi: 10.1155/2019/8719060 31885563 PMC6914872

[124] Samsu N. Diabetic nephropathy: challenges in pathogenesis. Diagnosis Treatment BioMed Res Int. (2021) 2021:1497449. doi: 10.1155/2021/1497449 34307650 PMC8285185

[125] Mortuza R Chen S Feng B Sen S Chakrabarti S. High glucose induced alteration of SIRTs in endothelial cells causes rapid aging in a p300 and FOXO regulated pathway. PloS One. (2013) 8:e54514. doi: 10.1371/journal.pone.0054514 23342163 PMC3546959

[126] Yasuda I Hasegawa K Sakamaki Y Muraoka H Kawaguchi T Kusahana E. Pre-emptive short-term nicotinamide mononucleotide treatment in a mouse model of diabetic nephropathy. J Am Soc Nephrol. (2021) 32:1355–70. doi: 10.1681/ASN.2020081188 PMC8259649 33795425

[127] Wang S Yang Y He X Yang L Wang J Xia S. Cdk5-mediated phosphorylation of sirt1 contributes to podocyte mitochondrial dysfunction in diabetic nephropathy. Antioxid Redox Signal. (2021) 34:171–90. doi: 10.1089/ars.2020.8038 32660255

[128] Zhang T Chi Y Kang Y Lu H Niu H Liu W. Resveratrol ameliorates podocyte damage in diabetic mice via SIRT1/PGC-1α mediated attenuation of mitochondrial oxidative stress. J Cell Physiol. (2019) 234:5033–43. doi: 10.1002/jcp.v234.4 30187480

[129] Xu Y Nie L Yin YG Tang JL Zhou JY Li DD. Resveratrol protects against hyperglycemia-induced oxidative damage to mitochondria by activating SIRT1 in rat mesangial cells. Toxicol Appl Pharmacol. (2012) 259:395–401. doi: 10.1016/j.taap.2011.09.028 22015446

[130] Li X Li Y Li F Chen Q Zhao Z Liu X. NAD(+) anabolism disturbance causes glomerular mesangial cell injury in diabetic nephropathy. Int J Mol Sci. (2022) 23(7):3458. doi: 10.3390/ijms23073458 35408818 PMC8998683

[131] Zhou L Xu DY Sha WG Shen L Lu GY Yin X. High glucose induces renal tubular epithelial injury via Sirt1/NF-kappaB/microR-29/Keap1 signal pathway. J Transl Med. (2015) 13:352. doi: 10.1186/s12967-015-0710-y 26552447 PMC4640239

[132] Sun X Huang K Haiming X Lin Z Yang Y Zhang M. Connexin 43 prevents the progression of diabetic renal tubulointerstitial fibrosis by regulating the SIRT1-HIF-1α signaling pathway. Clin Sci (Lond). (2020) 134:1573–92. doi: 10.1042/CS20200171 32558900

[133] Ren H Shao Y Wu C Lv C Zhou Y Wang Q. VASH-1 regulates oxidative stress and fibrosis in diabetic kidney disease via SIRT1/HIF1α and TGFβ1/smad3 signaling pathways. Front Mol Biosci. (2020) 7:137. doi: 10.3389/fmolb.2020.00137 32754616 PMC7365843

[134] Tikoo K Tripathi DN Kabra DG Sharma V Gaikwad AB. Intermittent fasting prevents the progression of type I diabetic nephropathy in rats and changes the expression of Sir2 and p53. FEBS Lett. (2007) 581:1071–8. doi: 10.1016/j.febslet.2007.02.006 17316625

[135] Liu Z Liu H Xiao L Liu G Sun L He L. STC-1 ameliorates renal injury in diabetic nephropathy by inhibiting the expression of BNIP3 through the AMPK/SIRT3 pathway. Lab Invest. (2019) 99:684–97. doi: 10.1038/s41374-018-0176-7 30683904

[136] Tang C Livingston MJ Liu Z Dong Z. Autophagy in kidney homeostasis and disease. Nat Rev Nephrol. (2020) 16:489–508. doi: 10.1038/s41581-020-0309-2 32704047 PMC7868042

[137] Feng J Lu C Dai Q Sheng J Xu M. SIRT3 facilitates amniotic fluid stem cells to repair diabetic nephropathy through protecting mitochondrial homeostasis by modulation of mitophagy. Cell Physiol Biochem. (2018) 46:1508–24. doi: 10.1159/000489194 29689547

[138] Jiao X Li Y Zhang T Liu M Chi Y. Role of Sirtuin3 in high glucose-induced apoptosis in renal tubular epithelial cells. Biochem Biophys Res Commun. (2016) 480:387–93. doi: 10.1016/j.bbrc.2016.10.060 27773814

[139] Locatelli M Zoja C Zanchi C Corna D Villa S Bolognini S. Manipulating Sirtuin 3 pathway ameliorates renal damage in experimental diabetes. Sci Rep. (2020) 10:8418. doi: 10.1038/s41598-020-65423-0 32439965 PMC7242337

[140] Wang XX Edelstein MH Gafter U Qiu L Luo Y Dobrinskikh E. G protein-coupled bile acid receptor TGR5 activation inhibits kidney disease in obesity and diabetes. J Am Soc Nephrol. (2016) 27:1362–78. doi: 10.1681/ASN.2014121271 PMC4849814 26424786

[141] Zhou Y Liu L Jin B Wu Y Xu L Chang X. Metrnl alleviates lipid accumulation by modulating mitochondrial homeostasis in diabetic nephropathy. Diabetes. (2023) 72:611–26. doi: 10.2337/db22-0680 PMC10130489 36812572

[142] Shi JX Wang QJ Li H Huang Q. SIRT4 overexpression protects against diabetic nephropathy by inhibiting podocyte apoptosis. Exp Ther Med. (2017) 13:342–8. doi: 10.3892/etm.2016.3938 PMC5245066 28123512

[143] Liu S Gao X Fan Z Wang Q. SIRT2 affects cell proliferation and apoptosis by suppressing the level of autophagy in renal podocytes. Dis Markers. (2022) 2022:4586198. doi: 10.1155/2022/4586198 35493297 PMC9054447

[144] Davizon-Castillo P McMahon B Aguila S Bark D Ashworth K Allawzi A. TNF-α-driven inflammation and mitochondrial dysfunction define the platelet hyperreactivity of aging. Blood. (2019) 134:727–40. doi: 10.1182/blood.2019000200 PMC6716075 31311815

[145] Bian C Zhang R Wang Y Li J Song Y Guo D. Sirtuin 6 affects glucose reabsorption and gluconeogenesis in type 1 diabetes via FoxO1. Mol Cell Endocrinol. (2022) 547:111597. doi: 10.1016/j.mce.2022.111597 35157928

[146] Han W Wang C Yang Z Mu L Wu M Chen N. SRT1720 retards renal fibrosis via inhibition of HIF1α/GLUT1 in diabetic nephropathy. J Endocrinol. (2019) JOE-18-0536.R2. doi: 10.1530/JOE-18-0536 30798323

[147] Liu M Liang K Zhen J Zhou M Wang X Wang Z. Sirt6 deficiency exacerbates podocyte injury and proteinuria through targeting Notch signaling. Nat Commun. (2017) 8:413. doi: 10.1038/s41467-017-00498-4 28871079 PMC5583183

[148] Ji L Chen Y Wang H Zhang W He L Wu J. Overexpression of Sirt6 promotes M2 macrophage transformation, alleviating renal injury in diabetic nephropathy. Int J Oncol. (2019) 55:103–15. doi: 10.3892/ijo.2019.4800 PMC6561622 31115579

[149] Muraoka H Hasegawa K Sakamaki Y Minakuchi H Kawaguchi T Yasuda I. Role of nampt-sirt6 axis in renal proximal tubules in extracellular matrix deposition in diabetic nephropathy. Cell Rep. (2019) 27:199–212.e5. doi: 10.1016/j.celrep.2019.03.024 30943401

[150] Huang R Fu P Ma L. Kidney fibrosis: from mechanisms to therapeutic medicines. Signal Transduct Target Ther. (2023) 8:129. doi: 10.1038/s41392-023-01379-7 36932062 PMC10023808

[151] Ram Mohan SD Shashidhar KN Anjanappa R Chandrappa M. Estimation of fluoride and sirtuin1 in patients with diabetic nephropathy in kolar district of karnataka. India J Lab Physicians. (2022) 14:57–64. doi: 10.1055/s-0041-1732817 36186264 PMC9519268

[152] Harrison EH Walusimbi-Kisitu M. Properties and subcellular localization of myocardial fatty acyl-coenzyme A oxidase. Am J Physiol. (1988) 255:H441–5. doi: 10.1152/ajpheart.1988.255.3.H441 3414812

[153] He W Wang Y Zhang MZ You L Davis LS Fan H. Sirt1 activation protects the mouse renal medulla from oxidative injury. J Clin Invest. (2010) 120:1056–68. doi: 10.1172/JCI41563 PMC2846063 20335659

[154] Xia Y Deng J Zhou Q Shao X Yang X Sha M. Expression and significance of Sirt1 in renal allografts at the early stage of chronic renal allograft dysfunction. Transpl Immunol. (2018) 48:18–25. doi: 10.1016/j.trim.2018.02.006 29452170

[155] Vasko R Xavier S Chen J Lin CH Ratliff B Rabadi M. Endothelial sirtuin 1 deficiency perpetrates nephrosclerosis through downregulation of matrix metalloproteinase-14: relevance to fibrosis of vascular senescence. J Am Soc Nephrol. (2014) 25:276–91. doi: 10.1681/ASN.2013010069 PMC3904558 24136919

[156] Kalluri R Weinberg RA. The basics of epithelial-mesenchymal transition. J Clin Invest. (2009) 119:1420–8. doi: 10.1172/JCI39104 PMC2689101 19487818

[157] He FF You RY Ye C Lei CT Tang H Su H. Inhibition of SIRT2 alleviates fibroblast activation and renal tubulointerstitial fibrosis via MDM2. Cell Physiol Biochem. (2018) 46:451–60. doi: 10.1159/000488613 29614506

[158] Sundaresan NR Bindu S Pillai VB Samant S Pan Y Huang JY. SIRT3 blocks aging-associated tissue fibrosis in mice by deacetylating and activating glycogen synthase kinase 3β. Mol Cell Biol. (2015) 36:678–92. doi: 10.1128/MCB.00586-15 PMC4760222 26667039

[159] Kume S Uzu T Horiike K Chin-Kanasaki M Isshiki K Araki S. Calorie restriction enhances cell adaptation to hypoxia through Sirt1-dependent mitochondrial autophagy in mouse aged kidney. J Clin Invest. (2010) 120:1043–55. doi: 10.1172/JCI41376 PMC2846062 20335657

[160] Chen J Xavier S Moskowitz-Kassai E Chen R Lu CY Sanduski K. Cathepsin cleavage of sirtuin 1 in endothelial progenitor cells mediates stress-induced premature senescence. Am J Pathol. (2012) 180:973–83. doi: 10.1016/j.ajpath.2011.11.033 PMC3349894 22234173

[161] Mitchell SJ Martin-Montalvo A Mercken EM Palacios HH Ward TM Abulwerdi G. The SIRT1 activator SRT1720 extends lifespan and improves health of mice fed a standard diet. Cell Rep. (2014) 6:836–43. doi: 10.1016/j.celrep.2014.01.031 PMC4010117 24582957

[162] Zeng L Chen R Liang F Tsuchiya H Murai H Nakahashi T. Silent information regulator, Sirtuin 1, and age-related diseases. Geriatr Gerontol Int. (2009) 9:7–15. doi: 10.1111/j.1447-0594.2008.00504.x 19260974

[163] Braidy N Guillemin GJ Mansour H Chan-Ling T Poljak A Grant R. Age related changes in NAD+ metabolism oxidative stress and Sirt1 activity in wistar rats. PloS One. (2011) 6:e19194. doi: 10.1371/journal.pone.0019194 21541336 PMC3082551

[164] Jin Q Jhun BS Lee SH Lee J Pi Y Cho YH. Differential regulation of phosphatidylinositol 3-kinase/Akt, mitogen-activated protein kinase, and AMP-activated protein kinase pathways during menadione-induced oxidative stress in the kidney of young and old rats. Biochem Biophys Res Commun. (2004) 315:555–61. doi: 10.1016/j.bbrc.2004.01.093 14975736

[165] Lee N Kim DK Kim ES Park SJ Kwon JH Shin J. Comparative interactomes of SIRT6 and SIRT7: Implication of functional links to aging. Proteomics. (2014) 14:1610–22. doi: 10.1002/pmic.201400001 24782448

[166] Liu Z Shi B Wang Y Xu Q Gao H Ma J. Curcumin alleviates aristolochic acid nephropathy based on SIRT1/Nrf2/HO-1 signaling pathway. Toxicology. (2022) 479:153297. doi: 10.1016/j.tox.2022.153297 36037877

[167] Mao RW He SP Lan JG Zhu WZ. Honokiol ameliorates cisplatin-induced acute kidney injury via inhibition of mitochondrial fission. Br J Pharmacol. (2022) 179:3886–904. doi: 10.1111/bph.v179.14 35297042

[168] Liu T Yang Q Zhang X Qin R Shan W Zhang H. Quercetin alleviates kidney fibrosis by reducing renal tubular epithelial cell senescence through the SIRT1/PINK1/mitophagy axis. Life Sci. (2020) 257:118116. doi: 10.1016/j.lfs.2020.118116 32702447

[169] Huang X Shi Y Chen H Le R Gong X Xu K. Isoliquiritigenin prevents hyperglycemia-induced renal injuries by inhibiting inflammation and oxidative stress via SIRT1-dependent mechanism. Cell Death Dis. (2020) 11:1040. doi: 10.1038/s41419-020-03260-9 33288747 PMC7721869

[170] Alzahrani S Zaitone SA Said E El-Sherbiny M Ajwah S Alsharif SY. Protective effect of isoliquiritigenin on experimental diabetic nephropathy in rats: Impact on Sirt-1/NFκB balance and NLRP3 expression. Int Immunopharmacol. (2020) 87:106813. doi: 10.1016/j.intimp.2020.106813 32707499

[171] Chen DQ Chen L Guo Y Wu XQ Zhao TT Zhao HL. Poricoic acid A suppresses renal fibroblast activation and interstitial fibrosis in UUO rats via upregulating Sirt3 and promoting β-catenin K49 deacetylation. Acta Pharmacol Sin. (2023) 44:1038–50. doi: 10.1038/s41401-022-01026-x PMC10104829 36470978

[172] Wang X Gao Y Tian N Wang T Shi Y Xu J. Astragaloside IV inhibits glucose-induced epithelial-mesenchymal transition of podocytes through autophagy enhancement via the SIRT-NF-κB p65 axis. Sci Rep. (2019) 9:323. doi: 10.1038/s41598-018-36911-1 30674969 PMC6344540

[173] Zhang Y Connelly KA Thai K Wu X Kapus A Kepecs D. Sirtuin 1 activation reduces transforming growth factor-β1-induced fibrogenesis and affords organ protection in a model of progressive, experimental kidney and associated cardiac disease. Am J Pathol. (2017) 187:80–90. doi: 10.1016/j.ajpath.2016.09.016 27993241

[174] Jin J Li W Wang T Park BH Park SK Kang KP. Loss of proximal tubular sirtuin 6 aggravates unilateral ureteral obstruction-induced tubulointerstitial inflammation and fibrosis by regulation of β-catenin acetylation. Cells. (2022) 11(9):1477. doi: 10.3390/cells11091477 35563783 PMC9100256

[175] Ye T Yang X Liu H Lv P Lu H Jiang K. Theaflavin protects against oxalate calcium-induced kidney oxidative stress injury via upregulation of SIRT1. Int J Biol Sci. (2021) 17:1050–60. doi: 10.7150/ijbs.57160 PMC8040307 33867828

[176] Lu S Zhou S Chen J Zheng J Ren J Qi P. Quercetin nanoparticle ameliorates lipopolysaccharide-triggered renal inflammatory impairment by regulation of sirt1/NF-KB pathway. J BioMed Nanotechnol. (2021) 17:230–41. doi: 10.1166/jbn.2021.3031 33785094

[177] Xiong W Xiong Z Song A Lei C Ye C Su H. UCP1 alleviates renal interstitial fibrosis progression through oxidative stress pathway mediated by SIRT3 protein stability. J Transl Med. (2023) 21:521. doi: 10.1186/s12967-023-04376-0 37533052 PMC10399010

[178] Zhou Z Qi J Kim JW You MJ Lim CW Kim B. AK-1, a Sirt2 inhibitor, alleviates carbon tetrachloride-induced hepatotoxicity *in vivo* and *in vitro* . Toxicol Mech Methods. (2020) 30:324–35. doi: 10.1080/15376516.2020.1729915 32063085

